# A machine learning model for early risk stratification of 28-day mortality after myocardial infarction

**DOI:** 10.1093/ehjdh/ztag079

**Published:** 2026-07-06

**Authors:** Pierre G Aublin, Stephanie G Kühne, David Füller, Benjamin Sasko, Hannah Schulze, Oliver Ritter, Ferdinand Bauke, Christine Meisinger, Jakob Linseisen, Philip Raake, Sebastian Zaunseder, Timo Schmitz, Dario Bongiovanni

**Affiliations:** Chair for Diagnostic Sensing, University of Augsburg, Eichleitnerstraße 30, 86159, Augsburg, Germany; Department of Internal Medicine I, Cardiology, University Hospital Augsburg, University of Augsburg, Stenglingstraße 2, 86156 Augsburg, Germany; Department of Internal Medicine I, Cardiology, University Hospital Augsburg, University of Augsburg, Stenglingstraße 2, 86156 Augsburg, Germany; Division of Cardiology, Department of Internal Medicine, University Hospital Brandenburg an der Havel, Brandenburg Medical School Theodor Fontane, Brandenburg an der Havel, Germany; Division of Cardiology, Department of Internal Medicine, University Hospital Brandenburg an der Havel, Brandenburg Medical School Theodor Fontane, Brandenburg an der Havel, Germany; Medical Department II, Ruhr University Bochum, Marien Hospital Herne, Herne, Germany; Division of Cardiology, Department of Internal Medicine, University Hospital Brandenburg an der Havel, Brandenburg Medical School Theodor Fontane, Brandenburg an der Havel, Germany; Division of Cardiology, Department of Internal Medicine, University Hospital Brandenburg an der Havel, Brandenburg Medical School Theodor Fontane, Brandenburg an der Havel, Germany; Department of Internal Medicine I, Cardiology, University Hospital Augsburg, University of Augsburg, Stenglingstraße 2, 86156 Augsburg, Germany; Institute of Epidemiology, Medical Faculty, University of Augsburg, Augsburg, Germany; Institute of Epidemiology, Medical Faculty, University of Augsburg, Augsburg, Germany; Department of Internal Medicine I, Cardiology, University Hospital Augsburg, University of Augsburg, Stenglingstraße 2, 86156 Augsburg, Germany; Chair for Diagnostic Sensing, University of Augsburg, Eichleitnerstraße 30, 86159, Augsburg, Germany; Institute of Epidemiology, Medical Faculty, University of Augsburg, Augsburg, Germany; Department of Internal Medicine I, Cardiology, University Hospital Augsburg, University of Augsburg, Stenglingstraße 2, 86156 Augsburg, Germany

**Keywords:** Mortality, Myocardial infarction, Risk score

## Abstract

**Aims:**

Despite significant improvement in transcatheter therapies, myocardial infarction (MI) retains a high burden of morbidity and mortality. While several scores have been developed, an established risk model tailored to predict short-term mortality after MI is lacking.

**Methods and results:**

In this study, we leveraged real-world data to develop a machine learning (ML) model for predicting 28-day mortality after MI, relying on clinical data collected within the first 24 h of hospitalization. From the Augsburg Myocardial Infarction Registry, 14 725 MI patients surviving the first 24 h of the acute event were included: 80% in the training set for model development to train a Gradient Boosting Machine while the 20% remaining served as test set. A sequential feature selection process retained variables as long as their addition improved AUPRC within 5-fold cross-validation on the train set. We then assessed our model on the test set and performed an external validation on 1328 patients from the Brandenburg Myocardial Infarction Registry. Shapley additive explanations values were used to explain the ML model’s predictions. Our model retained six variables including age, systolic blood pressure, admission levels of glucose, creatinine, leucocytes, and whether the patient had a prehospital cardiac arrest. It exhibited good calibration and discrimination ability on the internal (AUROC=0.82) and external validation (AUROC=0.85) and outperformed common scores like GRACE, CADILLAC, and TIMI (*P* = 0.05).

**Conclusion:**

The results highlight the potential of our model to ease the detection of patients at high risk, allowing targeted early intervention to improve their outcome, and to contribute to improved precision medicine.

## Graphical Abstract

**Graphical Abstract:**
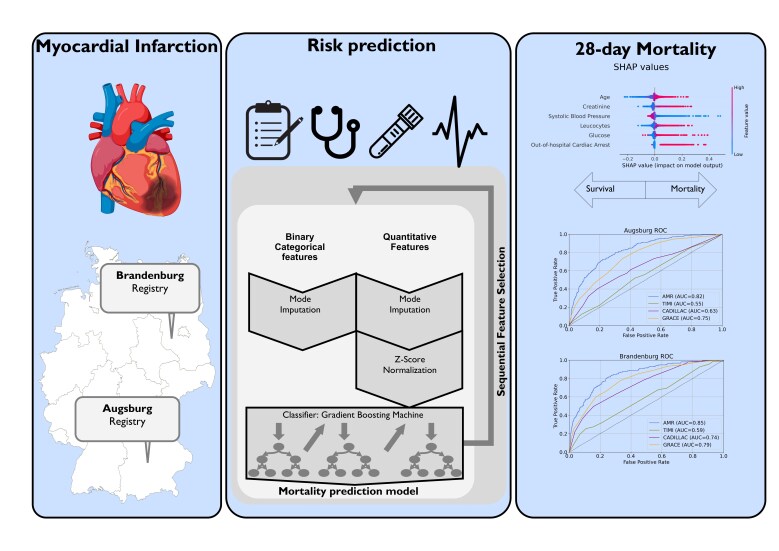

## Introduction

Despite advances in the treatment and prevention, acute myocardial infarction (MI) remains the global leading cause of morbidity and mortality.^1^ Early detection of patients at risk is vital to guide treatments and enable efficient resource allocation or tighter patient follow-up.

Large cohort studies have provided valuable insights into the characteristics associated to cardiovascular disease and its outcomes.^2,3^ Traditional risk scores such as TIMI, GRACE, or CADILLAC have been used to estimate short-term mortality after MI.^3–7^ However, these models were developed two decades ago, largely based on North American populations and show limited positive predictive value, which restricts their use in outcome prediction and precision medicine.

Machine learning (ML) approaches have shown promise in refining MI mortality discrimination in more complex NSTEMI cases, outperforming classical risk scores in terms of area under the receiver operating characteristic (AUROC).^8,9^ Similar results have been obtained on at-risk MI cases, including ICU patients developing cardiogenic shock.^10^ Incorporating quantitative biomarkers related to physiological signals or laboratory values could improve the predictions.^10,11^

However, many proposed models lack interpretability and show limited generalizability, which hinders their integration into clinical practice.^7^ Proposed ML models often also use more variables than traditional risk scores and it is not rare to see published approaches with at least 15 input variables. These models can also include parameters like left ventricular ejection fraction or medication, which are only available after MI treatment has been initiated, potentially reflecting local practice in the development cohort rather than the patient’s physiological state.^10–12^ Employing many input variables complicates routine use of the ML models as it then requires systematic collection of additional data—introducing an additional burden—and increases the risk of applying the models in the presence of missing variables. Accordingly, the current unmet need is not simply another high-performing mortality model, but a tool that combines strong predictive performance with parsimony, interpretability, external validation, and practical deployability in routine care.

In this study, we developed and externally validated a 24-h landmark model to predict 28-day mortality after MI in patients who survived the first 24 h of the acute event. The model relied only on variables that are routinely available at or shortly after admission and was externally validated in an independent registry.

## Methods

### Datasets

We used the regional Augsburg Myocardial Infarction Registry to train and develop our model.^13^ This registry provides comprehensive data from the region of Augsburg and operates using a population-based approach with complete enrolment. Myocardial infarction was included only if patients had their primary residence within the study area (approximately 705 000 people) and are 25–74 (until 2008) and 25–84 (2009 until 2019) years old, respectively. The registry included only hospitalized MI patients that survived the first 24 h of the acute event, and we applied no additional exclusion. Details on data collection and methodology can be found in prior publications.^13,14^ The dataset considered for this model includes comorbidities, symptoms, therapies, medication, and mortality for MI in the region of Augsburg.

We validated the model externally on the regional Brandenburg myocardial infarction registry, using the same information within the region of Brandenburg.^15,16^ This registry includes all patients presenting to the Emergency Department of the University Hospital of Brandenburg with a final diagnosis of acute myocardial infarction according to the Fourth universal definition of myocardial infarction.^17^ To align the external validation cohort with the 24-h landmark setting of the training cohort, we excluded patients of the Brandenburg MI registry who died within the first 24 h of the acute event. A flow diagram of the study population is provided in the Supplementary material online, *Figure S1* to describe the data constraints inherent to the registry.

### Inclusion criteria and primary endpoint

The primary endpoint of this study was 28-day all-cause mortality after the incident MI. We included all patients recorded in the respective registries with a diagnosis of acute MI (ST-segment elevation or non–ST-segment elevation) who were hospitalized during the study periods. Patients without documented 28-day mortality were excluded. After applying these criteria, 14 725 patients from the Augsburg registry (events between 2000 and 2019) and 1328 patients from the Brandenburg registry (events between 2019 and 2024) were available for analysis.

### Regulatory aspects and ethical approval

Ethical approval was provided by local authorities (Augsburg Myocardial infarction registry: Bavarian medical council Nr. 12057; Brandenburg registry Ethics Committee of Brandenburg Medical School Nr. E-01-20200923). The datasets provided to the authors were anonymized.

### Data availability

The raw data supporting the conclusions of this article are not publicly shared. The data will be made available by the corresponding authors with the consent of those responsible for the data set in Brandenburg and Augsburg upon reasonable request.

### Data pre-processing

**Figure 1:**
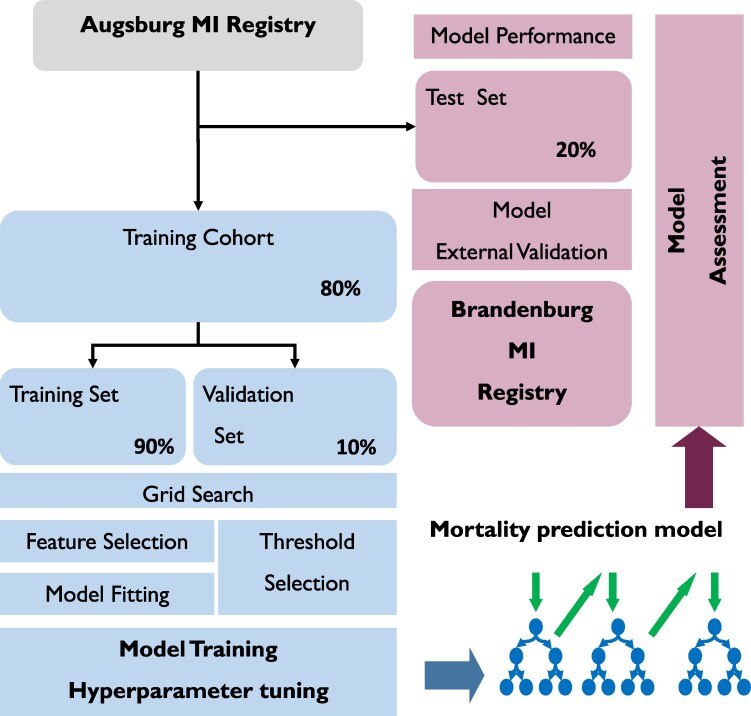
Schematic of the data workflow. 80% of the Augsburg MI registry served as training cohort for developing the model, while 20% served as internal validation cohort. The training cohort was split into a 90% training set and 10% held-out subset for hyperparameter optimization and decision threshold calibration. Finally, the MI model has been tested on the Brandenburg MI registry, which serves as external validation cohort.

The Augsburg registry was split into an 80% training and 20% test cohort, stratified by age, and year of the MI to account for the practice changes over time (*Figure 1*). Within the training cohort, 90% of the data served as a training set for fitting the model, while 10% were kept as a validation set for optimizing the algorithms’ hyperparameters and the final model decision threshold.

The model was trained using only objective data available at admission, excluding variables that would require patients’ self-report, and including demographic characteristics, information from the initial anamnesis and examination, and ECG findings. We also incorporated quantitative variables from the first blood sample obtained within 24 h of hospitalization. We intentionally excluded treatment strategy as a candidate predictor. As treatment allocation reflects both baseline severity and local or temporal practice patterns, including it could have reduced the transportability of the model across cohorts.

Missing variables were handled via mode imputation, which used the most frequent value, estimated on the training set, for quantitative features and the most frequent category for categorical features. While more advanced imputation methods such as multiple imputation may better account for uncertainty in missing data, they require more modelling assumptions and increase the computational complexity, particularly when introduced within feature selection procedures. As we wanted to develop an easily deployable model, we prioritized pragmatic imputation strategies. Although mode imputation for continuous variables is unconventional, it was chosen to maintain a simple and consistent pre-processing pipeline across variable types. To assess the robustness of this approach, we performed sensitivity analyses using median and mean imputation for continuous variables. Model discrimination and calibration were then compared across imputation strategies in the internal and external validation cohorts. Quantitative features were then standardized to zero mean and unit-variance, with mean and standard deviation estimated on the training set. Blood measurement variables in the Brandenburg Registry were converted from SI units (mmol/L) to the conventional units (mg/dL) used in the Augsburg Registry.

### Reference risk scores

We compared our model with three reference risk scores TIMI,^4,18^ GRACE,^5^ and CADILLAC.^6^ Because some of these scores were originally developed in distinct subpopulations, they were converted to their corresponding published risk estimates, as defined in the original derivation study to allow comparison across the overall cohort.^3,4,6,18^ No recalibration or refitting of those reference scores was performed. Detailed derivation rules for reference risk score variables not directly available in the registries are provided in the Supplementary Methods.

### Model development

The backbone classifier used in this study was a gradient boosting machine optimized with an exponential loss function and a learning rate of 0.1. The model consisted of 100 regression trees, using mean squared error as the split criterion, with a minimum of 32 samples required to split an internal node and at least four samples per leaf. Estimator parameters were selected through grid search over the hyperparameter space described in Supplementary material online, *Table S1*, using the training set and maximizing the area under the precision-recall curve (AUPRC) on the validation set.

To enhance the model’s practical applicability, we aimed to reduce the number of required input features. Beginning with 14 candidate variables, we conducted a sequential backward feature elimination, removing features until a decrease in AUPRC above 0.005 was observed, as estimated by 5-fold cross-validation within the training set (*Figure 1*). The final model was then fitted on the training set using the retained features. The operating threshold was subsequently selected in the Augsburg validation set by maximizing the F1-score as a pragmatic balance between sensitivity and positive predictive value. This choice was intended to limit false-positive alerts while maintaining acceptable detection of high-risk patients but should not be interpreted as universally optimal for all clinical use cases. Alternative thresholds can be applied depending on the intended clinical use case which may emphasize either sensitivity or PPV.

### Statistical analysis and model explanations

Continuous variables are summarized as medians and 95% confidence interval (CI) while categorical variables are presented as counts and percentages. Statistical significance was defined as a *P*-value ≤0.05.

To describe the population of the study cohorts and contextualize the model performance, we compared population baseline characteristics between Augsburg and Brandenburg registry. Mann–Whitney *U* test was used to compare the quantitative variables. Fisher's exact test was used to compare binary categorical variables.

To assess the reliability of the model’s output probability estimates, calibration curves were generated for the validation cohorts using 10 quantile bins, each containing approximately equal numbers of samples to ensure stable estimates across the range of predicted probabilities. Slope and intercept of these curves were estimated using a bootstrap to build a 95% CI. No recalibration was performed on the Augsburg test cohort or Brandenburg cohort, as the aim was to evaluate the model’s generalization in its original form on unseen data.

We report model discrimination using the AUROC and area under the precision recall curve (AUPRC). Model performance was compared against established risk scores, including GRACE, TIMI, CADILLAC. Although GRACE 2.0 was not primarily aimed at 28-day mortality, this score is derived based on the same variables as the original GRACE score and incorporates polynomial terms to model non-linear interaction among the used predictors.^7^ We therefore performed an additional comparison of our model with GRACE 2.0 (see Supplementary material online, *Figures S5*–S8).

Differences of AUROC and AUPRC were estimated using a stratified paired bootstrap for which positive and negative samples were resampled separately to preserve the class imbalance. The 95% CIs were then built from the bootstrap distribution of the differences.

Clinical utility was additionally assessed by decision curve analysis; methodological details are provided in the Supplementary Methods. We also report threshold dependent metrics (sensitivity, specificity, PPV, F1-Score) on the test cohorts only for our classifier to provide additional insights on the model’s generalization and practical usability for binary classification. As GRACE, TIMI, CADILLAC produce continuous scores rather than binary prediction, we do not report those threshold dependent metrics as the threshold setting could bias the comparison.

To analyse the contribution of each feature to the model’s decisions, Shapley values were computed on the train set.^19^ Shapley values provide a local explanation for each individual prediction by quantifying how much each feature contributed to the final output. Aggregating these values in a bee swarm plot reveals broader patterns in feature influence, enabling us to infer whether a given predictor functions primarily as a risk factor or as a factor associated with improved survival.

Patients with missing 28-day mortality in the external validation were excluded from the primary analysis. Baseline characteristics of patients with and without available follow-up were compared to check for potential selection bias.

To assess temporal transportability, we performed a sensitivity analysis using a chronological split of the Augsburg registry. Cases from 2000 to 2014 were used for model development and internal validation, whereas cases from 2015 to 2019 were reserved as a held-out temporal test cohort. Performance metrics from this analysis are reported in Supplementary material online, *Table S2*. We also performed subgroup analyses stratified by age (≤66 vs. >66 years), sex, MI type (STEMI vs. NSTEMI), and presence or absence of out-of-hospital cardiac arrest (OHCA) to assess model performance across heterogeneous MI populations. A comparison of the model performance on patients treated conservatively or invasively was also performed to test the robustness of the model across different treatment strategies. Discrimination and calibration were evaluated within each subgroup in both cohorts. Results are reported in Supplementary material online, *Tables S3*  to  *S7* and *Figures S2* and *S3*.

All machine learning development and statistical analyses were conducted in Python using Scikit-Learn 1.5.2 and Pandas 2.2.3 libraries. A graphical user interface is deployed online through the following link: https://mimortalityapp.onrender.com. The machine learning model and a Python-based graphical user interface application to run it are made publicly available through a Git repository https://github.com/gapiau20/mimortalityapp.git.

## Results

### Population baseline

The Augsburg and Brandenburg registries contributed 14 725 and 1328 patients with acute MI, respectively (*Table 1*). Variable-level missingness for all candidate predictors in both Augsburg and Brandenburg MI registries is reported in Supplementary material online, *Table S10*. Missingness ranged from 0% to 63%. The 28-day mortality rate was 7% in Augsburg and 11% in Brandenburg (*P* < 10^−4^), which reflects data from the ‘German Heart Report 2025’.^20^ Comparisons between the two registries revealed significant differences across multiple variables, reflecting substantial differences in population composition and registries’ structures. Patients from Brandenburg registry were older and had more comorbidities. Prehospital cardiac arrest was more frequent in Brandenburg than in Augsburg (7% vs. 4%, *P* < 10^−4^), and patients in Brandenburg more often presented with angina pectoris, whereas sweating at presentation was less frequently documented (*Table 1*).

**Table 1:** Baseline characteristics

	Augsburg	Brandenburg	*P*-value
Demographics			
Inclusion	2000–2019	2019–2024	
Patients	14 725	1328	—
Males	*10 607/14 725 (72%)*	*921/1325* (*69%)*	*0*.*0518*
Age	*66 (41–83; 14 725)*	*70 (45–91; 1325)*	*<0*.*0001*
Comorbidities			
Hypertension	11 315/14 712 (76%)	991/1304 (75%)	0.4
Hyperlipidaemia	8671/14 705 (58%)	572/1228 (46%)	<0.0001
Active smoker	8546/13 048 (34%)	560/906 (61%)	<0.0001
Previous stroke	1285/14 713 (9%)	88/566 (15%)	<0.0001
Diabetes	4744/14 713 (32%)	440/1306 (33%)	0.03
Symptoms at admission data			
Out-of-hospital cardiac arrest	*639/13 091 (4%)*	*96/1301 (7%)*	*<0*.*0001*
Angina pectoris	998/13 801 (7%)	1130/1306 (86%)	<0.0001
Dyspnoea	7005/14 655 (47%)	695/1301 (53%)	0.0001
Sweating	6308/14 618 (43%)	268/1328 (20%)	<0.0001
Syncope	892/14 620 (6%)	103/1307 (7%)	0.01
Heart rate at admission (bpm)	*80 (50–131;14 328)*	*84 (50–140; 1268)*	*<0*.*0001*
Systolic blood pressure at admission (mmHg)	*140(85–200; 14 247)*	*146 (85–199; 1252)*	*0*.*06*
Diastolic blood pressure at admission (mmHg)	*80 (50–119; 13 862)*	*84 (50–122; 1252)*	*<0*.*0001*
Positive troponin	*7971/9035 (88%)*	*1256/1326 (94%)*	*<0*.*0001*
C-reactive protein (mg/dL)	*0.49 (0.04–16.40; 13 675)*	*0.46 (0.06–16.79; 1312)*	*0*.*02*
Glucose (mmol/L)	*7.4 (4.7–19.9; 14 056)*	*7.9 (4.9–20.4; 1282)*	*<0*.*0001*
Creatinine (mg/dL)	*1.0 (0.6–3.0; 11 362)*	*1.0 (0.6–2.8; 1319)*	*0*.*4*
Total cholesterol (mg/dL)	*195 (106–301; 5421)*	*180 (93–296; 944)*	*<0*.*0001*
Leucocytes (/nL)	*10.1 (5.1–21.2; 11 390)*	*9.9 (5.3–21.2, 1324)*	*0*.*6*
MI type			
STEMI	5227/14 007 (37%)	451/1323 (34%)	0.02
Anterior wall MI	5514/10 012 (55%)	583/1223 (47%)	<0.0001
Treatment			
Conservative treatment	2735/14 725 (18%)	111/1263 (8%)	<0.0001
PCI	9997/14 704 (67%)	1074/1262 (85%)	<0.0001
Aortocoronary bypass	2042/14 667 (13%)	86/1253 (6%)	<0.0001
Outcomes			
Left ventricular ejection fraction (LVEF) < 30%	2059/8270 (25%)	129/1138 (11%)	<0.0001
Cardiogenic shock	907/14 653 (6%)	82/211 (38%)	<0.0001
In-hospital stroke	55/9138 (0.6%)	5/205 (2%)	0.01
In-hospital bleeding	201/9136 (2%)	26/209 (12%)	<0.0001
28-day mortality	1015/14 725 (7%)	153/1328 (11%)	<0.0001

Italic lines correspond to the features included as input of the feature selection. Quantitative variables are reported as median (95% CI; total), categorical variables as number/total (percentage). Hyperlipidaemia was defined as a patient-reported history of elevated cholesterol or cholesterol-lowering treatment. Positive troponin was defined as troponin I or T values above the upper reference limits used in Augsburg university hospital (see Methods section).

Patients in the Augsburg registry, included from 2000 to 2019 were treated more conservatively than patients in Brandenburg (included in 2019 to 2024) (*P* < 0.001). In the year 2019, data from both registries were available and the proportion of conservative treatments is similar in both registries: 89/698 (11%) patients in Augsburg and 12/180 (6%) patients in Brandenburg (*P* = 0.07). There was a steady increase of interventional strategies over time on both registries (see Supplementary material online, *Figure S4*). The trends are similar in both registries and consistent with the broader shift towards invasive management of acute coronary syndromes.^21^

In the Brandenburg MI registry, 304/1632 patients (18%) had missing mortality data and were excluded from the primary analysis. Compared with patients with documented follow-up, those without follow-up had less documented hypertension and hyperlipidaemia and were more often active smokers, whereas admission vital signs, laboratory values, MI type, and treatment patterns were broadly similar (see Supplementary material online, *Table S8*).

### Model and performance

**Figure 2:**
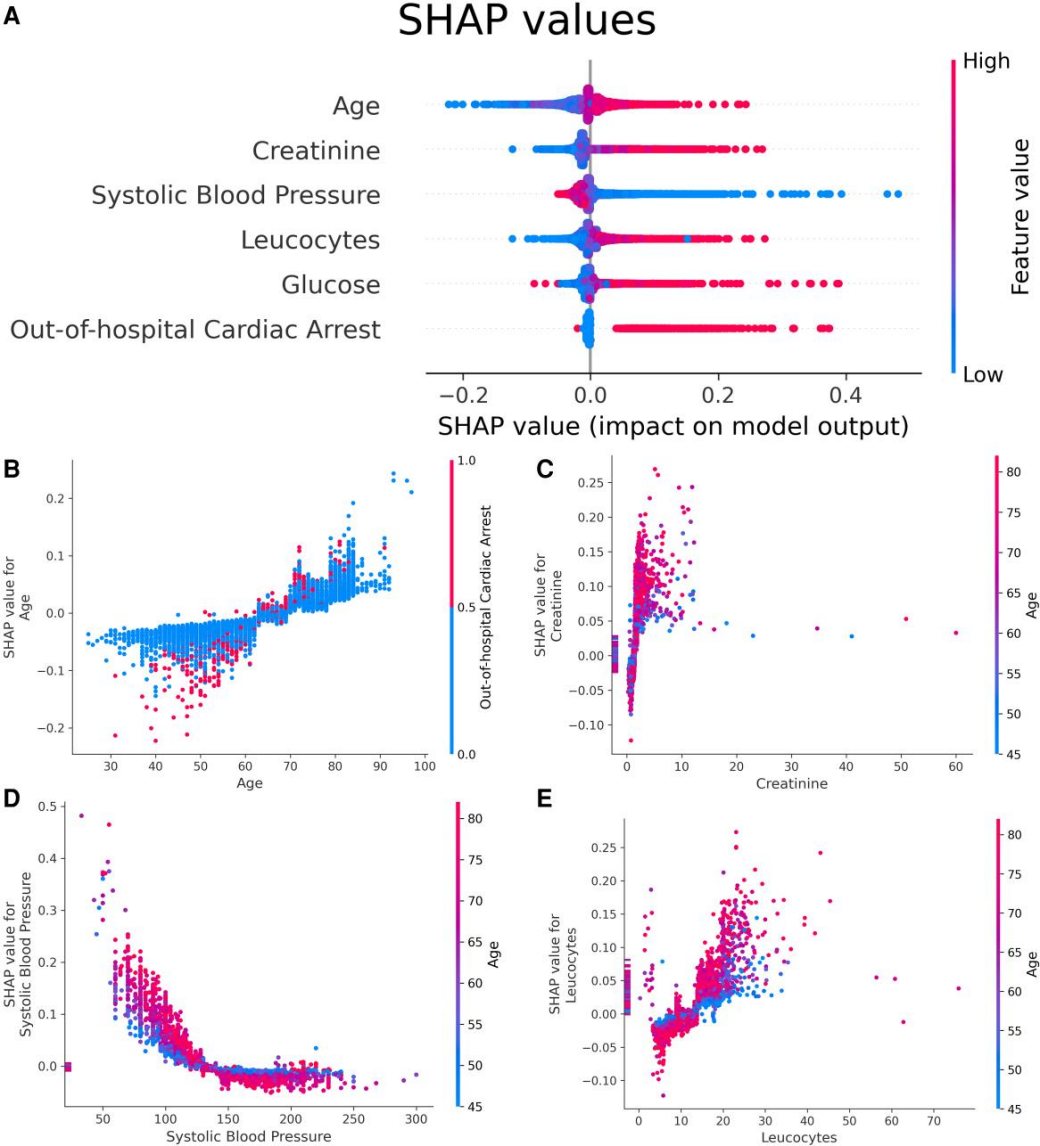
Variables selected in our model. Shapley values on the training set (*A*). Subplots (*B*), (*C*), (*D*), (*E*) highlight the interaction of SHAP values between different features.

The final model retained six predictors available at or shortly after admission: out-of-hospital cardiac arrest, age, serum creatinine, systolic blood pressure at admission, leucocyte count, and plasma glucose (*Figure 2A*). Higher age, lower systolic blood pressure, and higher admission levels of glucose, creatinine, and leucocytes were associated with increased predicted mortality risk. Patients who had suffered an out-of-hospital cardiac arrest were consistently classified as high-risk by the model. Dependence plots showed that the impact of these laboratory and clinical variables on risk became more pronounced at older ages (*Figure 2 B–E*).

In the Augsburg test cohort, the model achieved at the operating threshold a sensitivity of 0.41 (95% CI 0.34–0.48), specificity of 0.94 (0.93–0.95), positive predictive value (PPV) of 0.33 (0.26–0.38), and F1-score of 0.36 (0.30–0.42). In the Brandenburg cohort, sensitivity was higher, 0.49 (0.41–0.57), with a specificity of 0.93 (0.91–0.94), PPV of 0.47 (0.40–0.55) and F1-score of 0.48 (0.41–0.55), consistent with the greater mortality in this higher-risk population (*Table 2*).

**Table 2:** Classification performance (median, 95% CI) of the AMR model at operating threshold on the Augsburg and Brandenburg test cohorts

Metric	Augsburg test	Brandenburg
**Discrimination**		
Sensitivity	0.41 (0.34–0.48)	0.49 (0.41–0.57)
Specificity	0.94 (0.93–0.95)	0.93 (0.91–0.94)
PPV	0.33 (0.26–0.38)	0.47 (0.40–0.55)
F1-score	0.36 (0.30–0.42)	0.48 (0.41–0.55)
AUROC	0.82 (0.79–0.85)	0.85 (0.81–0.88)
AUPRC	0.28 (0.22–0.34)	0.47 (0.38–0.55)
**Calibration**		
Slope	0.91 (0.80–1.02)	1.07 (0.91–1.24)
Intercept	−0.12 (−0.44 to 0.16)	0.54 (0.17–0.92)
Brier score	0.06 (0.05–0.06)	0.08 (0.07–0.09)

**Figure 3:**
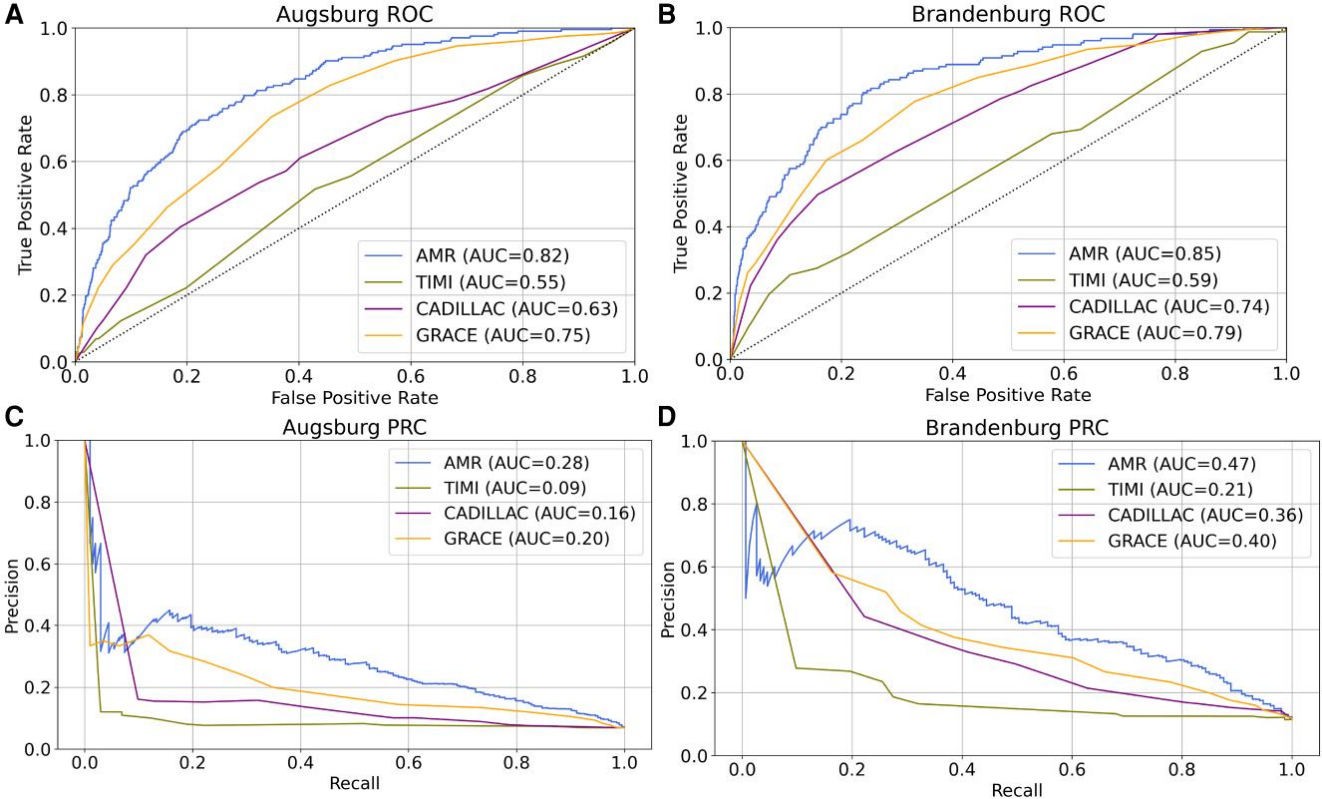
Receiver operating characteristic (*A*, *B*) and precision-recall curve (*C*, *D*) of our AMR model, GRACE, TIMI, CADILLAC scores on test set and Brandenburg MI registry.

Discrimination was good in both cohorts, with AUROC values of 0.82 (95% CI 0.79–0.85) in the Augsburg test cohort and 0.85 (0.81–0.88) in the Brandenburg registry cohort. The AUPRC was 0.28 (0.22–0.34) in the Augsburg test cohort and 0.47 (0.38–0.55) in the Brandenburg cohort, the higher value in the external cohort largely reflecting the higher event rate in that cohort (*Tables 1* and *2*, *Figure 3*).

**Figure 4:**
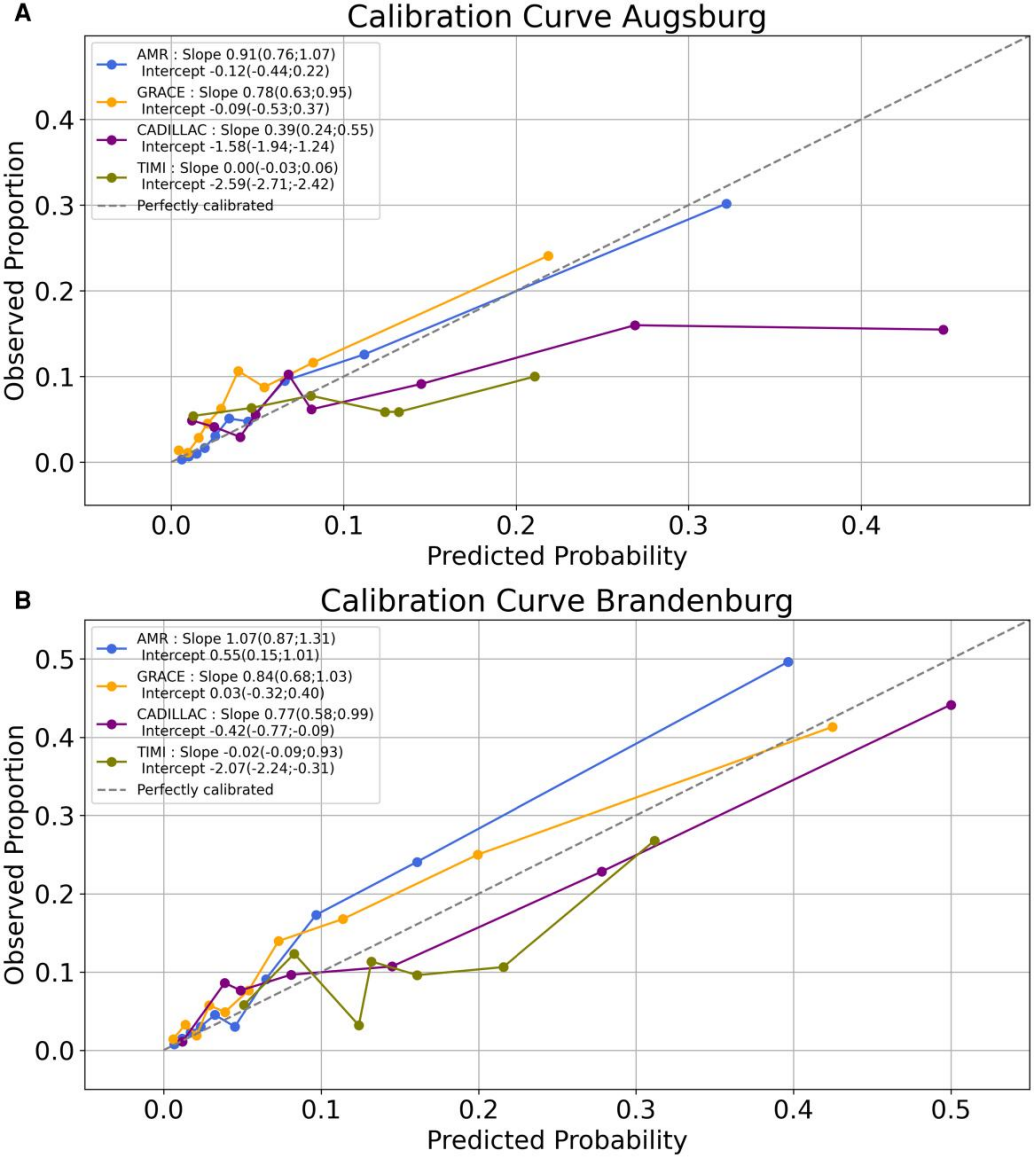
Calibration curve of the model against classical risk scores on the test set (*A*) and Brandenburg (*B*) MI registry. Slope and intercepts are reported as median (95% CI). A quantile-based binning was used to derive the calibration curves and therefore each bin contains an equal number of patients.

Calibration was good in the Augsburg test cohort, with a slope of 0.91 (95% CI 0.80 to 1.02) and an intercept of −0.12 (−0.44 to 0.16). In the Brandenburg cohort, the slope was 1.07 (0.91–1.24) and the intercept 0.54 (0.17–0.92), indicating underestimation of absolute risk in the external validation cohort. The Brier score was 0.06 (0.05–0.06) in Augsburg and 0.08 (0.07–0.09) in Brandenburg. However, this difference should be interpreted with caution given the higher mortality in Brandenburg cohort (*Table 1*, *Figure 4*).

Subgroup analyses showed that the model maintained broadly consistent discrimination across most patient subgroups in both cohorts (see Supplementary material online, *Table S3*–S7). Area under the receiver operating characteristic values were 0.86 and 0.88 in STEMI, 0.80 and 0.83 in NSTEMI, 0.84 and 0.85 in men, and 0.78 and 0.84 in women in Augsburg and Brandenburg, respectively. Lower discrimination was observed in older patients and in patients with OHCA, with AUROC values of 0.74 and 0.80 in patients aged >66 years and 0.68 and 0.79 in patients with OHCA. In contrast, AUROC values were 0.89 and 0.87 in younger patients and 0.80 and 0.81 in patients without OHCA. The model’s discrimination also dropped notably in patients treated conservatively (AUROC of 0.76 in Augsburg, and 0.79 in Brandenburg) compared to those treated with PCI or CABG (AUROC of 0.83 in Augsburg and 0.84 in Brandenburg). Sensitivity analyses showed that model performance remained broadly stable across mean, median, and mode imputation strategies. Discrimination and calibration metrics varied only modestly across approaches in both the internal and external validation cohorts. While mean and median imputation showed slightly higher performance in some settings, the overall differences were small, and the main predictor structure remained consistent (see Supplementary material online, *Figure S9* and Supplementary material online, *Table S11*).

### Comparison to reference scores

Our Augsburg Mortality Risk (AMR) model was well calibrated in the internal cohort with a slope and intercept close to 1 and 0, respectively, whereas in the Brandenburg dataset it tended to underestimate absolute risk, with a positive intercept although the slope remained close to 1 (Supplementary material online, *Tables S2* and *S4*). On the latter cohort, all models tended to underestimate the mortality risk with calibration curves above the perfect calibration line (observed proportion = predicted probability).

**Figure 5:**
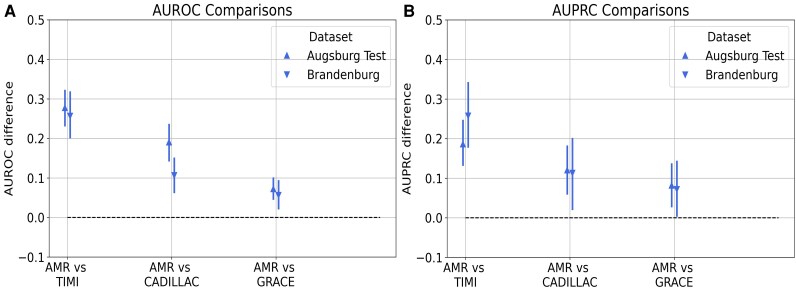
Bootstrap bar plots (95% CI) of the AUROC and AUPRC differences on the test set (Augsburg test) and Brandenburg MI registry (Brandenburg). A positive difference means the AUROC or AUPRC is higher for our AMR model than the compared score.

In both validation cohorts, our model achieved higher discrimination than the three established risk scores. The AUROC of our model was significantly higher than that of TIMI, GRACE as well as CADILLAC. The same pattern was observed in the external cohort (*P* < 0.05 for all pairwise comparisons) for AUPRC, indicating a significantly better identification of true high-risk patients under class imbalance (*Figures 3* and *5*).

**Figure 6:**
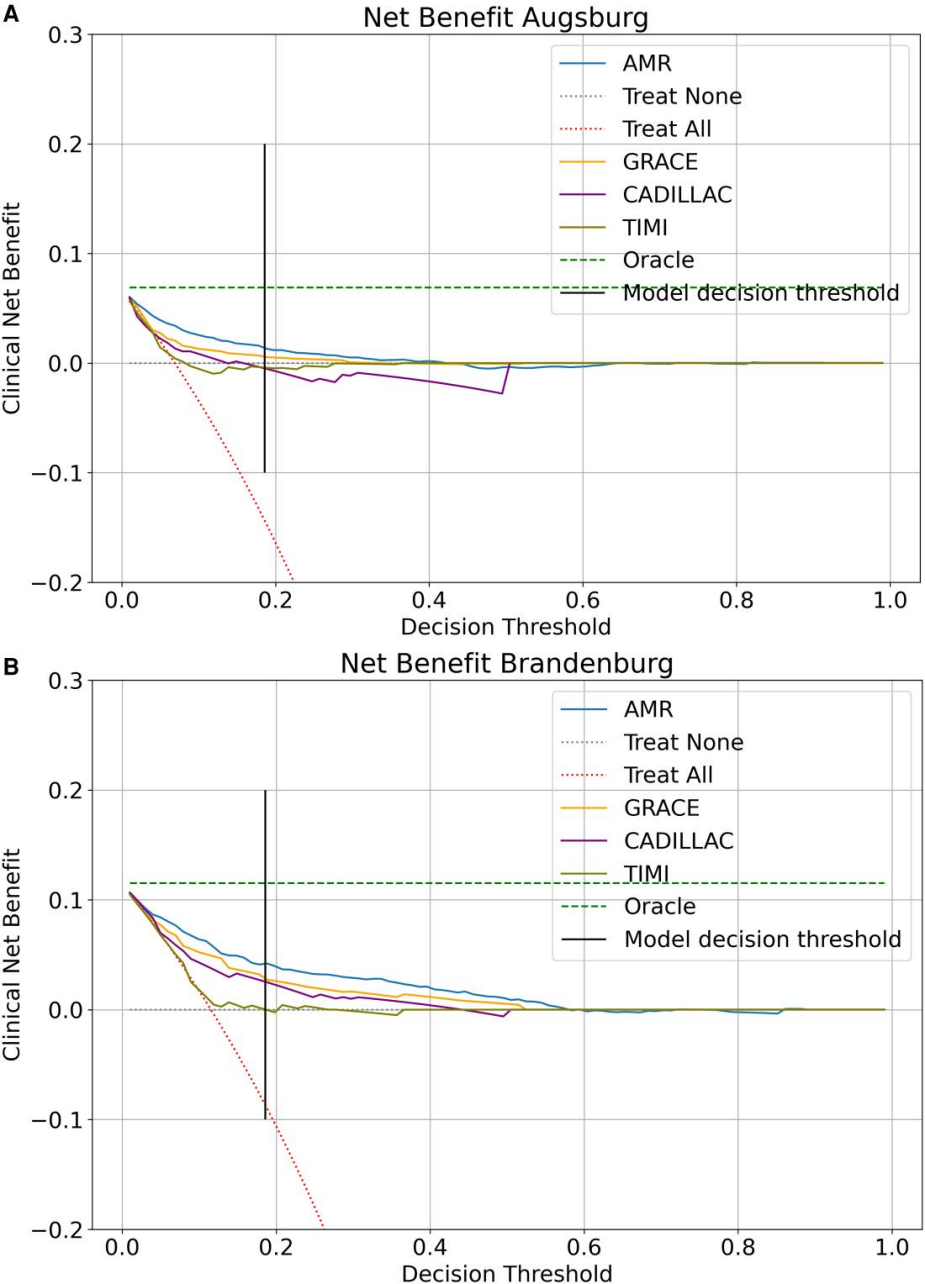
Net clinical benefit across the decision thresholds in the test set (*A*) and Brandenburg MI registry (*B*). Curves compare the AMR model against GRACE, TIMI, CADILLAC scores, and default treat-all and treat-none strategies. The vertical black line indicates the model decision threshold of the AMR model adjusted on the Augsburg Validation Set.

In decision curve analysis, the net benefit of the AMR model was consistently higher than that of TIMI, GRACE, and CADILLAC across clinically plausible thresholds (*Figure 6*).

## Discussion

In this study, we developed and externally validated a new machine learning model for predicting 28-day mortality after acute MI that relies on only six routinely available variables at or shortly after admission.

Compared with the established TIMI,^4,18^ CADILLAC,^6^ GRACE,^5^ and even GRACE 2.0^7^ scores, our model consistently achieved higher AUROC and AUPRC values, indicating superior discrimination and improved PPV. The predictors retained by the feature selection are well-established risk factors in cardiovascular medicine, and our goal was not to identify novel variables. Rather, the contribution of our approach lies in showing that a compact set of routinely available early predictors can achieve robust performance, external validity, and practical usability in a contemporary real-world setting. Compared with TIMI, CADILLAC, GRACE, and GRACE 2.0, our model achieved significantly higher AUROC and AUPRC values despite relying on fewer and more readily available inputs.^4,6,18^ The model showed broadly consistent performance across subgroups defined by age, sex, MI type, and OHCA status. Lower discrimination in older patients and in patients with OHCA likely reflects greater clinical heterogeneity, whereas the absence of major differences between STEMI and NSTEMI supports the robustness of the selected predictors across MI presentations.

These gains are particularly relevant in a setting where short-term mortality after MI has declined towards around 7% in many European regions.^22^ Improved PPV may enhance the clinical usefulness of the model by reducing false-positive alerts in low-event settings.^16,23^ Decision curve analysis showed positive net benefit of the AMR model at the selected operating threshold and generally higher net benefit than comparator scores across clinically plausible thresholds (*Figure 6*). In this setting, a positive classification may support closer monitoring or closer early follow-up. The F1-based threshold should be viewed as a pragmatic default. Depending on the intended clinical application, alternative thresholds may be preferable to prioritize either sensitivity or positive predictive value and should therefore be adapted before local deployment.

Our findings extend prior work on classical risk scores in acute coronary syndromes, which were developed more than two decades ago using logistic regression and broader variable sets and have since been widely implemented for bedside risk stratification and benchmarking of outcomes. In our contemporary cohorts with high use of guideline-directed therapies, the TIMI, GRACE, and CADILLAC scores showed suboptimal discrimination. This pattern is consistent with reports that temporal improvements in MI care and outcomes may reduce the calibration performance of legacy scores if they are not updated or re-fitted to contemporary populations.^24–26^

A relevant limitation of the classical risk scores is their reliance on logistic regression.^3,4,6^ This approach is straightforward and allows the derivation of simple point-based scores from the weights or odds ratios. However, logistic regression models assume linear and additive relationships between the variables. Shapley values on the other hand can provide insights into the contribution of each variable to the individual model predictions. By aggregating the Shapley values across the training data, we could obtain a more flexible view of the interaction between the different model variables, in comparison to the fixed weights of the traditional risk scores. Our SHAP analyses showed that age, initial creatinine level, and systolic blood pressure had the largest impact on the predicted risk. While these three factors, as well as OHCA, are also embedded within the GRACE score, our model highlights non-linear interactions between those four variables as shown on the dependence plots (*Figure 2B–E*).^5^ These findings are consistent with pathophysiological understanding and with earlier registry data from the MONICA/KORA region and other cohorts, where older age, impaired renal function and cardiogenic shock were key determinants of short-term mortality.^27,28^ Interestingly, age modulates the other variables’ weight, amplifying the effects of OHCA, hypotension and inflammatory response (*Figure 2B–D*).^17^ Elevated admission glucose is a biologically plausible predictor of short-term mortality after myocardial infarction, reflecting both pre-existing dysglycaemia and an acute stress response to the ischaemic event. It may therefore serve as an early marker of infarct severity and haemodynamic compromise.^29–31^ Because the AMR model is based on a gradient boosting machine, it cannot be reduced to simple cut-off values for individual predictors. SHAP analyses support interpretability, but they do not define universal thresholds. We therefore provide an online calculator and source code for individualized risk estimation.

A recent update of the GRACE score (GRACE 3.0) relying on a large cohort was proposed to address sex-specific differences of the GRACE score. Nevertheless, GRACE 3.0 has been developed accounting only for variables selected in previous score versions and improving the score restricting it only to these variables. Our model integrated a range of clinical variables that are routinely available and often directly present in registry data, as reflected in Augsburg and Brandenburg MI registries, and therefore follows a different approach.

Moreover, GRACE 3.0 scores were targeted towards NSTEMI patients.^32,33^ Although we included the type of MI for the feature selection, this variable was not retained by the feature selection in the final model. This would suggest that either the information regarding the MI type was not an independent predictor of mortality on the Augsburg cohort^34^ or that the information about this feature could be captured through other variables. Our model can be applied without requiring prior MI classification or ECG interpretation. Currently, GRACE 3.0 is only available through an online calculator^35^ and does not allow an automated application on large cohorts. We were therefore not able to test and compare it to our model.

In our model, we reduced the number of input features to six variables available shortly upon admission. This approach mitigates the challenge of collecting a large number of clinical variables as seen in prior models.^8,11,12^ Importantly, our model only incorporates features independent from patient-reported symptoms. This is particularly advantageous as patients with MI present with a broad spectrum of symptoms, some of which they may fail to describe accurately.^21,36^ Such omissions can indeed lead to incorrect triage or diagnostic delays, potentially hindering timely treatment initiation. Treatment strategy was not included in the model because it is influenced by both baseline risk and local practice patterns. The lower discrimination observed in conservatively treated patients suggests greater heterogeneity in this subgroup and should be considered when interpreting transportability.^10,37^ Hence, the novelty of our study lies less in proposing mortality prediction itself than in developing a parsimonious, explainable, externally validated, and directly deployable digital tool for early post-admission risk stratification after myocardial infarction.

The developed model relied on mode imputation, which provided a simple and uniform pre-processing strategy but is unconventional for continuous variables. Sensitivity analyses using mean and median imputation showed broadly comparable discrimination and calibration, suggesting that the main findings were robust to the imputation strategy. We therefore retained mode imputation for the primary model because it supported a simpler and slightly more parsimonious pipeline.

The external validation was performed on another independent population-based registry from a distinct German region with more recent data compared to the Augsburg registry. The substantial differences between Augsburg and Brandenburg indicate a pronounced case-mix shift. Regional differences in post-MI mortality within Germany are well documented,^16,20^ and we therefore consider it a strength that the model maintained acceptable discrimination in an external cohort with a markedly different baseline risk profile. At the same time, the systematic underestimation of absolute risk in Brandenburg shows that relative risk ranking transported better than absolute probability estimates and that local recalibration is likely necessary before implementation. The reference scores were not recalibrated to the Augsburg or Brandenburg cohorts, which may have disadvantaged these comparator models and may have widened part of the observed gap, particularly for calibration. However, because all models, including AMR, were evaluated without local adaptation, the comparison reflects their relative out-of-sample transportability.

The validation cohort had a relevant proportion of missing 28-day mortality data (18%). Patients without follow-up differed in some baseline characteristics, suggesting that some selection bias cannot be excluded and that model performance in the external validation cohort may be somewhat overestimated.

This study has some limitations. The validation was performed only on German registries. Nevertheless, while regional differences could be observed both in terms of population characteristics, outcomes and in terms of treatment, our model outperformed reference scores also in the external validation cohort. The registries used were also developed over slightly different timeframes. The Augsburg registry contains data until 2019. It therefore remains unclear whether there were time-specific effects, particularly related to COVID-19 within the period 2020–2023 that might explain part of the mortality difference between both population registries.^38–40^ A temporal sensitivity analysis using a chronological split of the Augsburg registry showed broadly stable discrimination across earlier and later calendar periods, supporting the robustness of the overall predictive framework despite evolving practice over time (see Supplementary material online, *Table S2*). However, calibration results show that the models developed on earlier data tend to overestimate the risk in patients treated more recently, highlighting the need for recalibration of the models as practice evolves. In this analysis, CRP was selected instead of leukocyte count, whereas the other retained predictors remained unchanged. A further restriction of model development to only the most recent years was not feasible because of the limited number of events.

A further limitation is that Killip classwas not directly available in the registries and had to be approximated from the available variables for calculation of the reference risk scores. Because this approach does not fully correspond to the standard clinical definition, it may have introduced imprecision into the comparison with legacy scores. This limitation concerns the comparator scores only and did not affect development of the AMR model.

As a 24-h landmark model, our tool is intended to support early post-admission risk stratification after initial stabilization and laboratory assessment, rather than immediate triage at presentation. In this setting, a high predicted risk may support closer monitoring or prolonged observation in a monitored unit, whereas a low predicted risk may contribute to more streamlined care pathways and discharge planning in otherwise stable patients. Any such use should be viewed as decision support complementing clinical judgment, and prospective studies are needed before routine implementation. As patients who died within the first 24 h were not represented in the training cohort, survivor bias cannot be excluded, and performance should be interpreted with caution when using the model outside this setting.

## Conclusion

We developed a compact and explainable machine learning model that is superior in terms of short-term mortality prediction after MI compared with several classical risk scores using only six easily available and routinely collected admission variables. Before wider implementation, local recalibration and further external validation are needed to ensure accurate risk estimation across populations with different baseline risk profiles. Future research may also explore dynamic model extensions and integration into digital clinical decision support systems.

## Supplementary Material

ztag079_Supplementary_Data
