# Supplementary material for: A machine learning model for early risk stratification of 28-day mortality after myocardial infarction
Source: Eur Heart J Digit Health. 2026 Jul 6;7(6):ztag079. doi: 10.1093/ehjdh/ztag079 (PMC13335799; doi:10.1093/ehjdh/ztag079)
Supplement: ztag079_Supplementary_Data [file ztag079_supplementary_data.docx]

# A machine learning model for early risk stratification of 28-day mortality after myocardial infarction

Supplementary material

Pierre G. Aublin^1,2^, Stephanie G. Kühne^2^, David Füller ^3^, Benjamin Sasko ^3,4^, Hannah Schulze^3^, Oliver Ritter^3^, Ferdinand Bauke ^2^, Christine Meisinger ^5^, Jakob Linseisen^5^, Philip Raake^2^, Sebastian Zaunseder^1 *^, Timo Schmitz ^5*^, Dario Bongiovanni^2 *^

1. University of Augsburg, Chair for Diagnostic Sensing, Germany
2. Department of Internal Medicine I, Cardiology, University Hospital Augsburg, University of Augsburg, Germany
3. Division of Cardiology, Department of Internal Medicine, University Hospital Brandenburg an der Havel, Brandenburg Medical School Theodor Fontane, Germany
4. Medical Department II, Ruhr University Bochum, Marien Hospital Herne, Herne, Germany
5. Institute of Epidemiology, University of Augsburg, Augsburg, Germany

* Equal contribution

# Index

[Supplementary Methods 3](#_Toc227573632)

[Reference risk scores derivation 3](#_Toc227573633)

[Decision Curve Analysis 4](#_Toc227573634)

[Supplementary Figures 5](#_Toc227573635)

[Cohort selection 5](#_Toc227573636)

[Subgroup Analyses: Bootstrap Comparisons 6](#_Toc227573637)

[Treatment trends over time 8](#_Toc227573638)

[GRACE 2.0 score 9](#_Toc227573639)

[Sensitivity to Imputation: SHAP Analyses 13](#_Toc227573640)

[Supplementary Tables 14](#_Toc227573641)

[Model hyperparameter tuning 14](#_Toc227573642)

[Temporal drift 15](#_Toc227573643)

[Subgroups sensitivity analyses 16](#_Toc227573644)

[Missing data and sensitivity analyses 19](#_Toc227573645)

[Sensitivity to imputation 22](#_Toc227573646)

[References 23](#_Toc227573647)

# Supplementary Methods

## Reference risk scores derivation

Reference Risk scores including TIMI, CADILLAC, GRACE (1 and 2) include some features that were not available within Augsburg and Brandenburg myocardial infarction (MI) registries. Those risk scores for MI patients include some features that were not primarily available within the Augsburg and Brandenburg registries. We derived these variables using the information present in the registries and subsequently derived the corresponding risk scores. Because these imputed variables and the resulting scores include information that would not be directly available at admission, they were not used as inputs for training the ML models. Instead, they were used exclusively for comparison of model performance in the test cohorts.

Killip class ^1^ at admission was derived using admission systolic blood pressure (SBP), admission heart rate and whether the patient had dyspnoea. Patients with hypotension (SBP<90) and tachycardia (HR>100 bpm), or who suffered a prehospital cardiac arrest were assigned to Killip IV. Patients reporting dyspnoea at admission and having tachycardia were assigned to Killip III. Patients with dyspnoea were assigned to Killip II, while the remaining patients were considered as Killip I.

For CADILLAC score computation, anaemia was defined as admission haemoglobin concentration below 13 g/dL for men and 12 g/dL for women. For TIMI score computation, a patient was considered to have positive cardiac enzymes if the admission troponin T concentration exceeded 0.014ng/mL or his admission troponin I concentration exceeded 0.034 ng/mL, as per local definition. Renal insufficiency was defined as an estimated glomerular filtration rate (eGFR) below 60 mL/min/1.73 m². In the Brandenburg MI registry, hypertension was assumed if prior to the MI, the patient was taking at least one of the following treatments: ACE Inhibitors, betablockers, hydrochlorothiazide, or calcium channel blockers. Hyperlipidaemia was defined as an LDL cholesterol level above 130 mg/dL or total cholesterol level above 200 mg/dL.

## Decision Curve Analysis

We performed decision curve analysis to evaluate the clinical utility of the model based on the net benefit as described by Vickers et al. (20). As shown in equation (1), a ‘net benefit’ was computed for different probability decision thresholds ($p_{t}$). This term represents the benefit of true-positive identifications and adds a penalty to capture the harm arising from false positive classifications. The decision curve of our model was then compared to the curves of different decision strategies, including treating all patients (treat all’) or treating no patient (treat none’) independently from the model decision threshold. In this context, treatment refers to classifying the patient as at risk of mortality within 28-day, and potentially initiating a corresponding clinical action based on this label. The method provides a graphical output that combines the model’s discrimination capabilities and calibration, hence providing insights on the decision-making utility of the model. Results of this additional analysis are presented in the supplementary material (Supplementary Figure 1).

| $\text{Net Benefit=}\frac{\text{True Positive Count}}{\text{n}}\text{-(}\frac{\text{p}_{\text{t}}}{\text{1-}\text{p}_{\text{t}}}\text{)}\frac{\text{False Positive Count}}{\text{n}}$ | (1) |
| --- | --- |

# Supplementary Figures

## Cohort selection


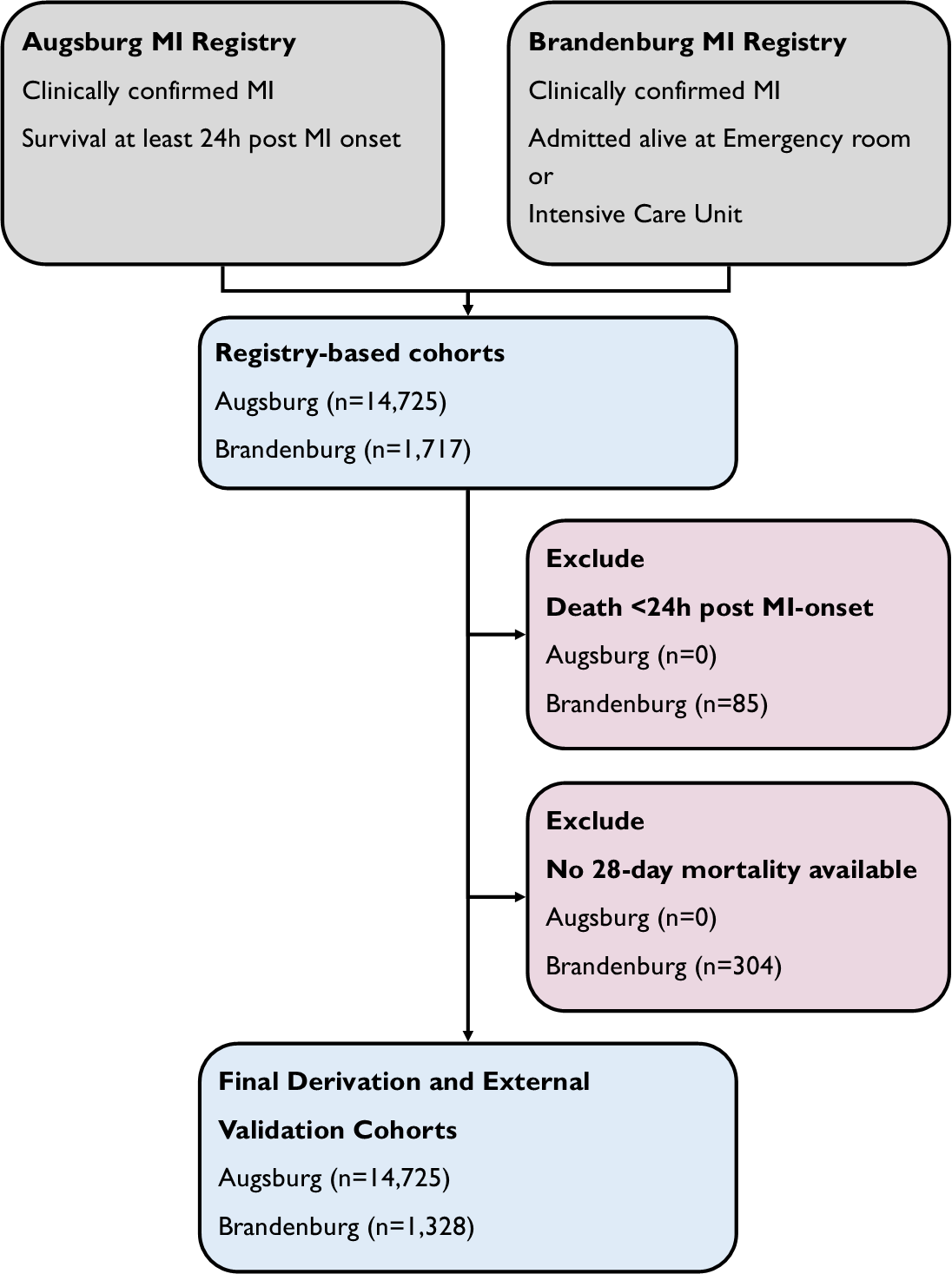


##### ***Supplementary Figure 1.*** *Flow diagram of the study population in the Augsburg and Brandenburg registries*

## Subgroup Analyses: Bootstrap Comparisons


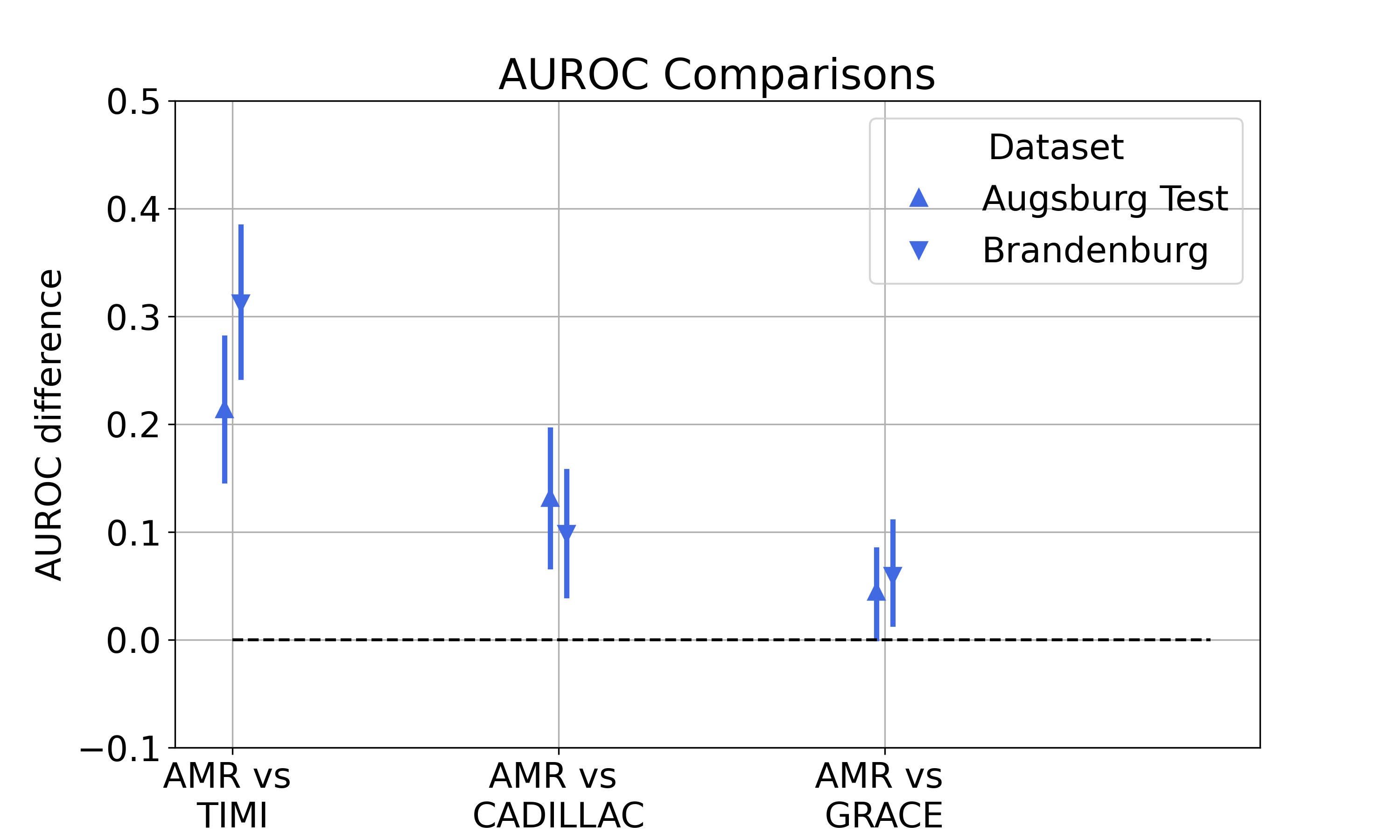


##### **Supplementary Figure 2.** AUROC differences (95%-CI) between the AMR model and reference scores in patients aged >66 years.


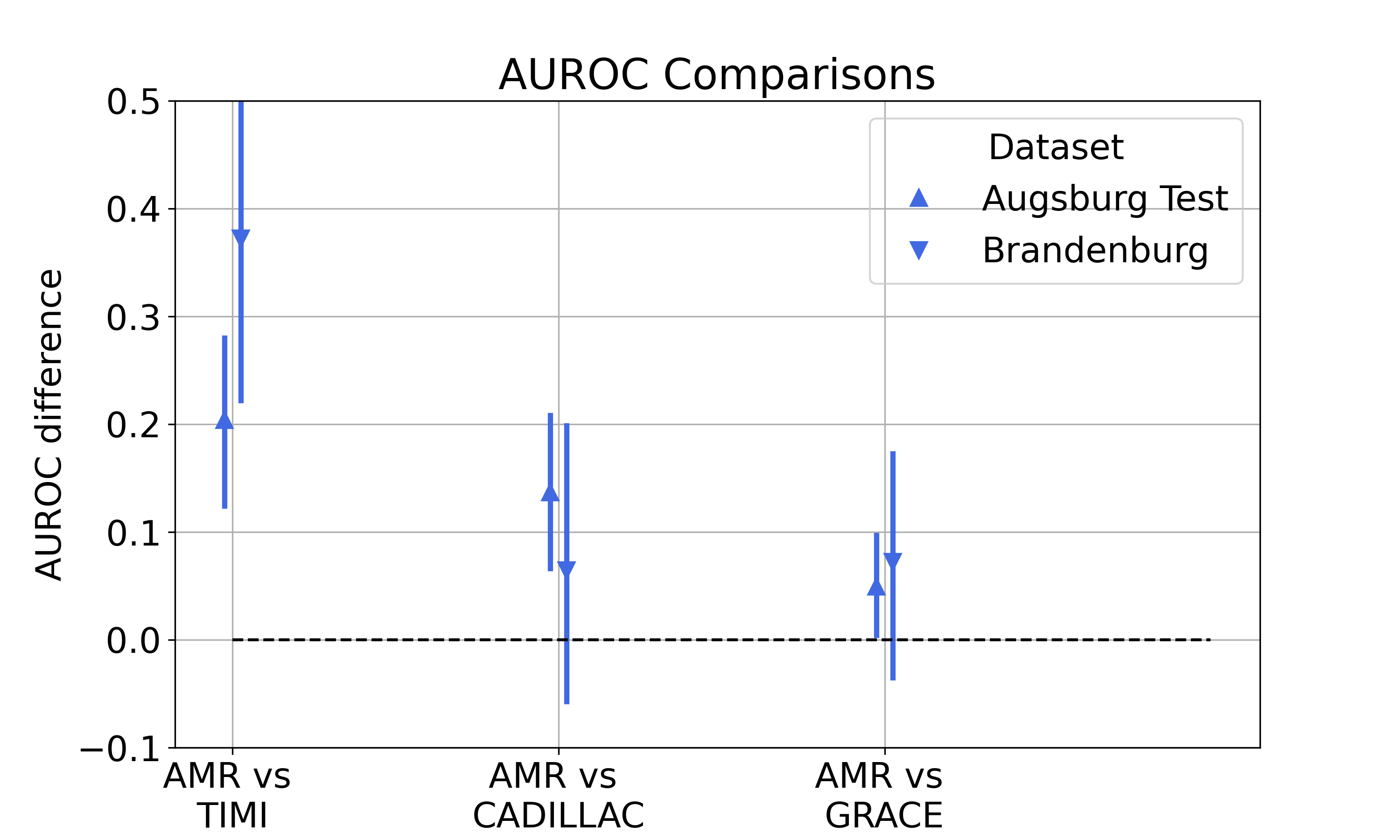


##### **Supplementary Figure 3.** AUROC differences between the AMR model and reference scores in conservatively treated patients. Error bars indicate 95% CI. A positive value indicates a higher AUROC for the AMR model.

## Treatment trends over time


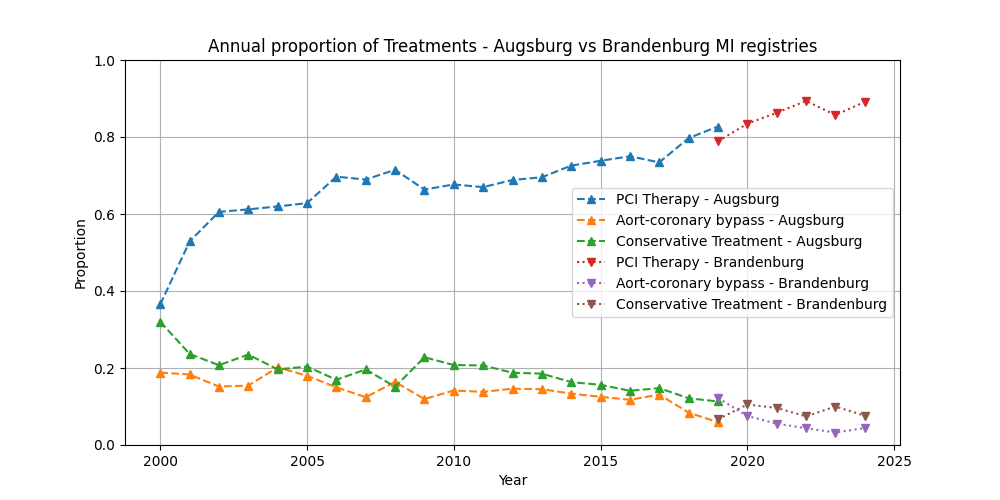


##### **Supplementary Figure 4.** Temporal trends in MI treatment strategies. Annual proportions of PCI, aorto-coronary bypass, and conservative treatment in the Augsburg and Brandenburg registries from 2000 to 2024.

## GRACE 2.0 score

The GRACE 2.0 score predicts 1-year mortality based on the same features as the original GRACE score. To provide a broader comparison with established risk models, we additionally report the results for GRACE 2.0 in this Supplementary Material. **
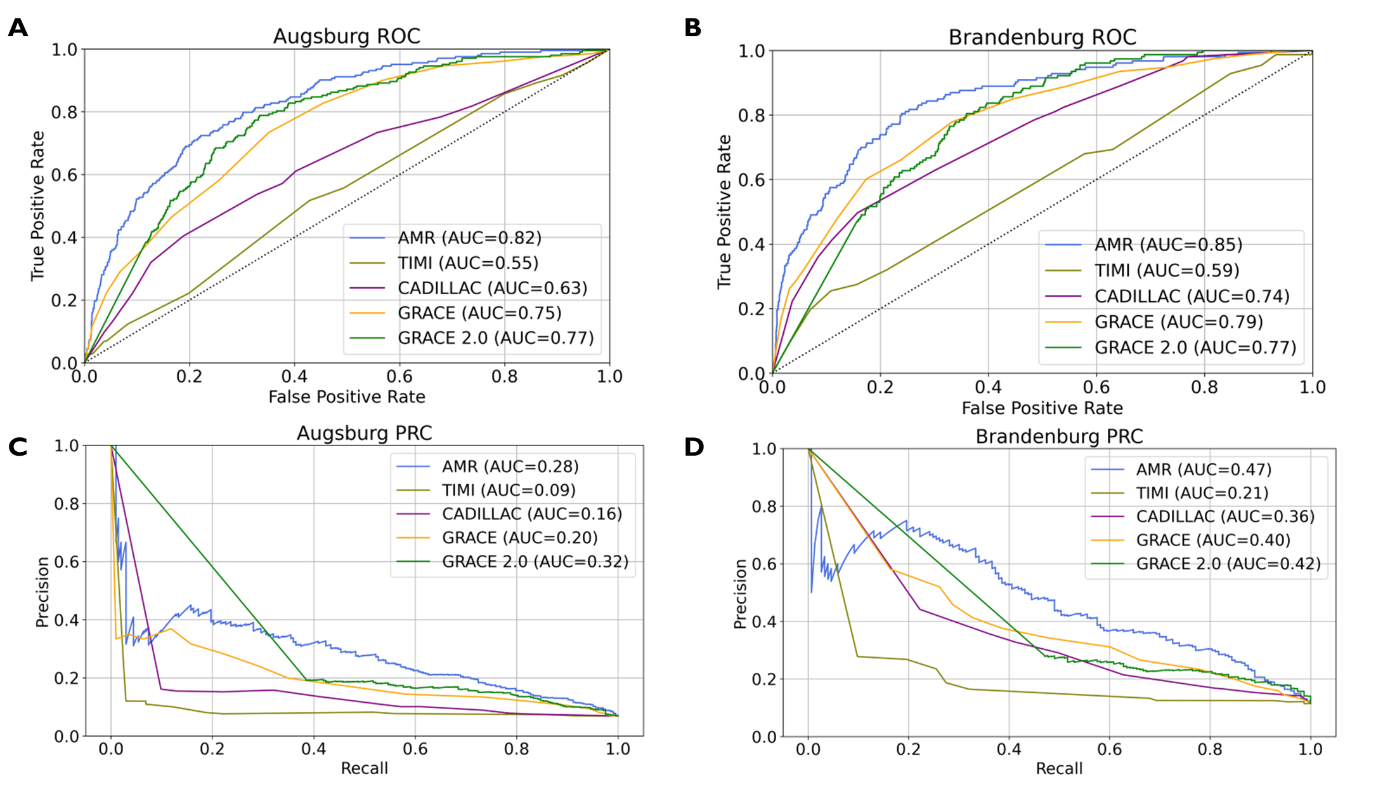
**

##### ***Supplementary Figure 5.*** *Receiver Operating Characteristic (A, B) and Precision-Recall Curves (C, D) of the AMR model, TIMI, CADILLAC and GRACE 1 and 2 scores in the test cohorts.*

#####
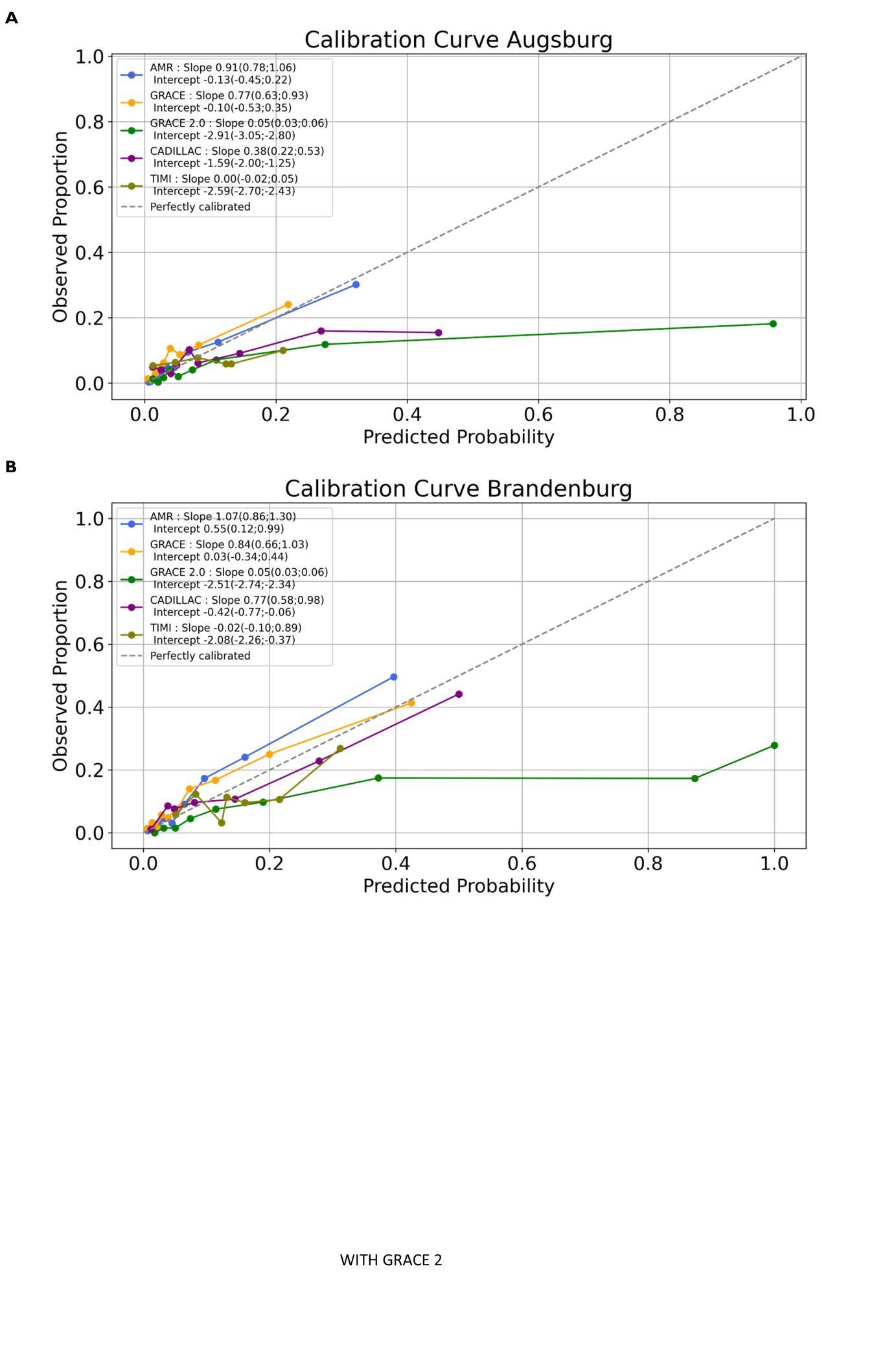
***Supplementary Figure 6.*** *Calibration curve of the AMR model and reference risk scores in the Augsburg (A), and Brandenburg (B) registries. Calibration slopes and intercepts are reported as median (95% CI).*


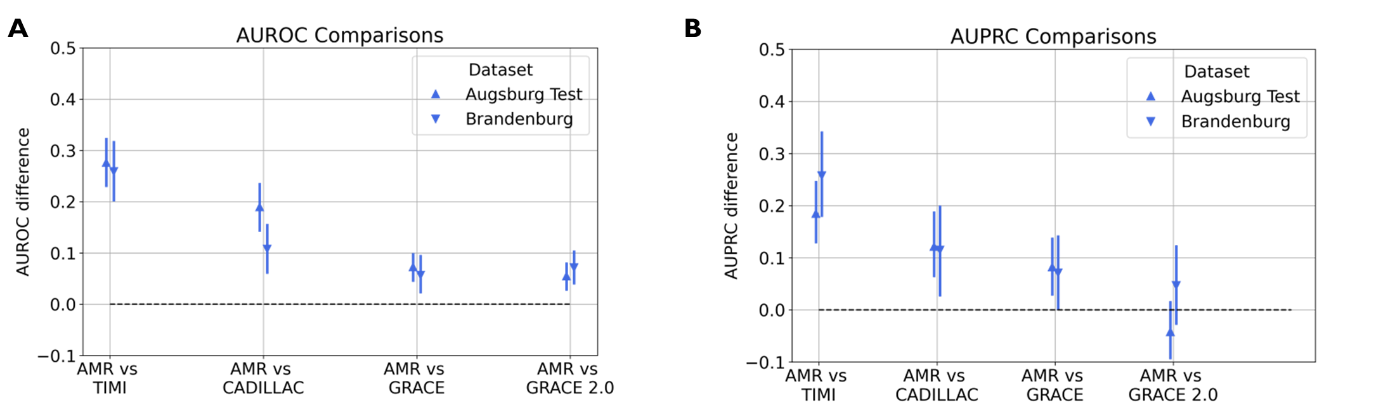


##### **Supplementary Figure 7.** Bootstrap bar plots (95% CI) of the AUROC and AUPRC differences. A positive difference indicates that the AMR model achieved a higher AUROC or AUPRC than the compared score. Augsburg Test: cohort from Augsburg registry excluded from model development and used only as test cohort.


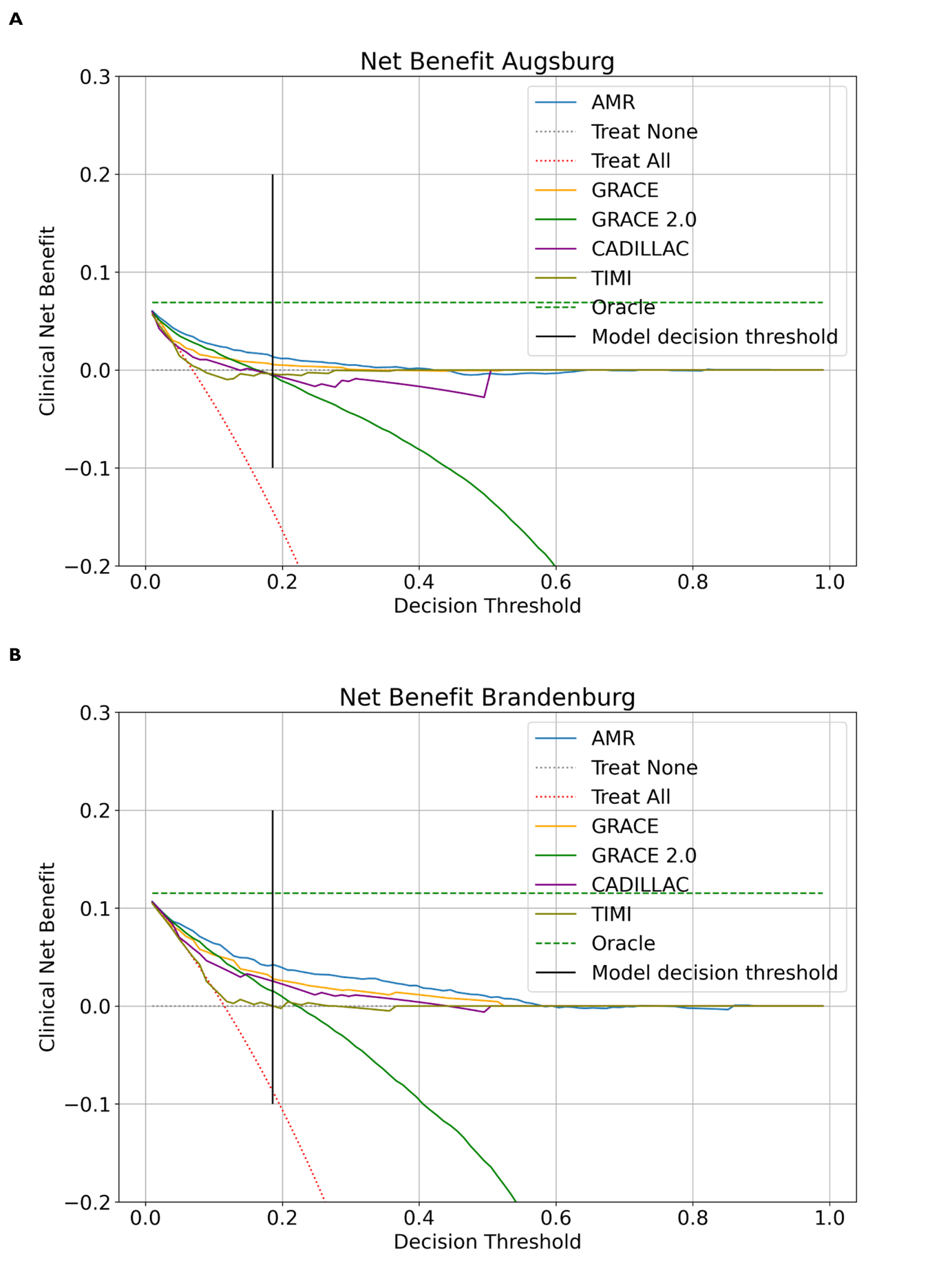


##### **Supplementary Figure 8.** Decision curve analysis in the Augsburg rest set (A) and Brandenburg cohort (B). Curves compare the AMR model with GRACE, TIMI, CADILLAC scores, as well as the treatment strategies. The vertical black line indicates the model decision threshold of the AMR model adjusted on the Augsburg Validation Set.

## Sensitivity to Imputation: SHAP Analyses


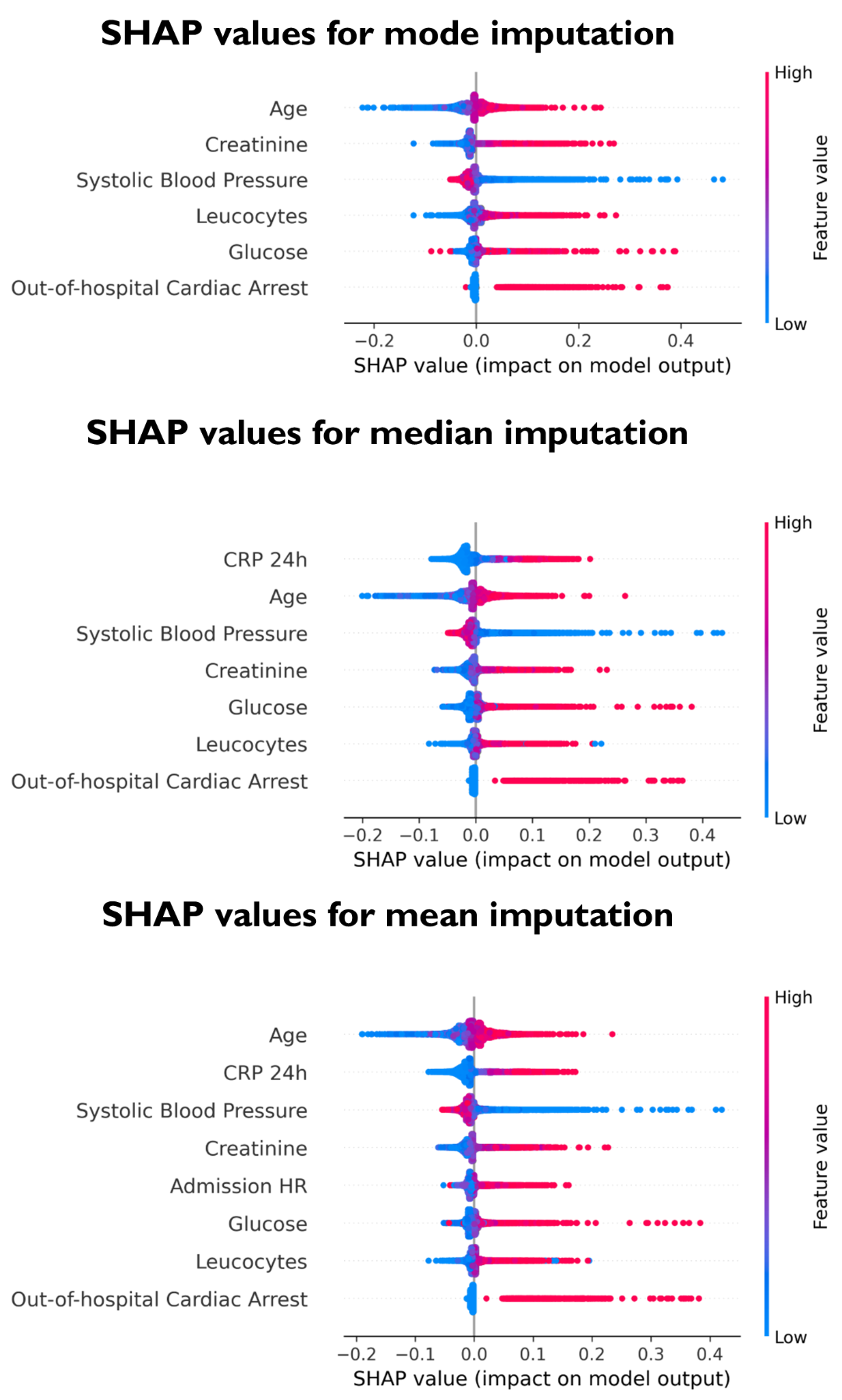


##### **Supplementary Figure 9.** SHAP summary plots for models trained using mode, median, mean imputation.

# Supplementary Tables

## Model hyperparameter tuning

##### **Supplementary Table *1.*** Hyperparameter space used for the grid-search optimization of the gradient boosting machine model.

| Boosting loss | Log-Loss, Exponential |
| --- | --- |
| Minimum samples for node split | 2,16,32 |
| Minimum samples per leaf | 2,16,32 |
| Learning rate | 0.1,0.05,0.01 |

## Temporal drift

To assess temporal transportability, we performed a chronological split of the Augsburg registry, using cases from 2000 to 2014 for model development and validation and cases from 2015 to 2019 as a held-out temporal test set.

The model retained 6 features: age, first measured glucose level, admission systolic blood pressure, first C-reactive protein measurement, first creatinine level, and out-of-hospital cardiac arrest.

##### **Supplementary Table 2.** Performance of the AMR model using a temporal split on the Augsburg registry between train and test set.

| **Selected features** | Age, Glucose, Systolic Blood Pressure, Out-of-hospital Cardiac Arrest, CRP 24h and Creatinine | | |
| --- | --- | --- | --- |
| **Model Performance** | **Augsburg  Validation (2000-2014)** | **Augsburg Test (2015-2019)** | **Brandenburg** |
| **Discrimination** |  |  |  |
| Sensitivity | 0.45 (0.34-0.57) | 0.39 (0.33-0.44) | 0.46 (0.38-0.54) |
| Specificity | 0.91 (0.90-0.93) | 0.93 (0.92-0.94) | 0.91 (0.89-0.93) |
| PPV | 0.28 (0.20-0.35) | 0.27 (0.23-0.31) | 0.40 (0.33-0.47) |
| F1-score | 0.34 (0.26-0.42) | 0.32 (0.27-0.36) | 0.43 (0.36-0.49) |
| AUROC | 0.84 (0.80-0.88) | 0.83 (0.80-0.85) | 0.81 (0.77-0.84) |
| AUPRC | 0.32 (0.22-0.44) | 0.26 (0.21-0.32) | 0.39 (0.31-0.47) |
| **Calibration** |  |  |  |
| Slope | 0.89 (0.73-1.06) | 0.83 (0.75-0.94) | 0.79 (0.66-0.94) |
| Intercept | -0.28 (-0.70-0.12) | -0.37 (-0.60--0.13) | 0.01 (-0.31-0.36) |
| Brier score | 0.05 (0.05-0.07) | 0.05 (0.05-0.06) | 0.09 (0.07-0.10) |

## Subgroups sensitivity analyses

##### **Supplementary Table 3.** Model performance by age group in the Augsburg and Brandenburg cohorts. Values are median (95% CI)

| **Metric\Subpopulation** | **Age****≤66 Augsburg** | **Age≤66 Brandenburg** | **Age >66 Augsburg** | **Age >66 Brandenburg** |
| --- | --- | --- | --- | --- |
| **N samples** | **1519** | **560** | **1426** | **765** |
| Sensitivity | 0.43 (0.32-0.54) | 0.44 (0.27-0.63) | 0.40 (0.31-0.48) | 0.50 (0.41-0.59) |
| Specificity | 0.97 (0.97-0.98) | 0.98 (0.96-0.99) | 0.90 (0.88-0.91) | 0.89 (0.86-0.91) |
| PPV | 0.47 (0.35-0.58) | 0.50 (0.30-0.69) | 0.27 (0.20-0.33) | 0.46 (0.37-0.55) |
| F1-score | 0.45 (0.34-0.55) | 0.47 (0.29-0.62) | 0.32 (0.25-0.39) | 0.48 (0.40-0.55) |
| AUROC | 0.89 (0.84-0.92) | 0.87 (0.78-0.94) | 0.74 (0.70-0.79) | 0.80 (0.75-0.84) |
| AUPRC | 0.40 (0.28-0.52) | 0.47 (0.27-0.68) | 0.24 (0.18-0.31) | 0.47 (0.37-0.56) |
| Calibration slope | 1.19 (1.00-1.41) | 1.16 (0.86-1.57) | 0.73 (0.59-0.89) | 0.99 (0.80-1.18) |
| Calibration intercept | 0.76 (0.17-1.37) | 0.59 (-0.37-1.50) | -0.57 (-0.96--0.21) | 0.44 (0.02-0.86) |
| Brier score | 0.04 (0.03-0.05) | 0.04 (0.02-0.05) | 0.08 (0.07-0.09) | 0.11 (0.09-0.13) |

##### **Supplementary Table 4.** Model performance by MI type in the Augsburg and Brandenburg cohort. Values are median (95% CI)

| **Metric\Subpopulation** | **STEMI Augsburg** | **STEMI Brandenburg** | **NSTEMI Augsburg** | **NSTEMI Brandenburg** |
| --- | --- | --- | --- | --- |
| **N samples** | **995** | **451** | **1823** | **872** |
| Sensitivity | 0.47 (0.35-0.59) | 0.60 (0.46-0.73) | 0.38 (0.30-0.46) | 0.43 (0.34-0.53) |
| Specificity | 0.94 (0.93-0.96) | 0.93 (0.90-0.95) | 0.93 (0.92-0.95) | 0.93 (0.91-0.95) |
| PPV | 0.34 (0.25-0.45) | 0.52 (0.39-0.64) | 0.31 (0.24-0.39) | 0.44 (0.35-0.54) |
| F1-score | 0.39 (0.30-0.49) | 0.55 (0.43-0.66) | 0.34 (0.27-0.41) | 0.44 (0.35-0.52) |
| AUROC | 0.86 (0.81-0.90) | 0.88 (0.83-0.92) | 0.80 (0.77-0.84) | 0.83 (0.78-0.87) |
| AUPRC | 0.28 (0.20-0.38) | 0.52 (0.38-0.67) | 0.28 (0.21-0.36) | 0.44 (0.33-0.54) |
| Calibration slope | 1.00 (0.82-1.23) | 1.08 (0.85-1.37) | 0.85 (0.72-0.99) | 1.05 (0.87-1.26) |
| Calibration intercept | -0.00 (-0.54-0.55) | 0.46 (-0.13-1.08) | -0.22 (-0.61-0.15) | 0.55 (0.12-1.05) |
| Brier score | 0.05 (0.04-0.06) | 0.07 (0.06-0.09) | 0.06 (0.05-0.07) | 0.08 (0.07-0.10) |

##### **Supplementary Table 5.** Model performance by sex and test cohort. Values are median (95% CI)

| **Metric\Subpopulation** | **Male Augsburg** | **Male Brandenburg** | **Female Augsburg** | **Female Brandenburg** |
| --- | --- | --- | --- | --- |
| **N samples** | **2120** | **921** | **825** | **404** |
| Sensitivity | 0.43 (0.35-0.51) | 0.51 (0.41-0.61) | 0.36 (0.25-0.49) | 0.46 (0.34-0.59) |
| Specificity | 0.94 (0.93-0.95) | 0.94 (0.92-0.96) | 0.94 (0.92-0.95) | 0.90 (0.86-0.93) |
| PPV | 0.33 (0.26-0.40) | 0.49 (0.39-0.59) | 0.31 (0.21-0.43) | 0.44 (0.31-0.57) |
| F1-score | 0.37 (0.30-0.44) | 0.50 (0.41-0.58) | 0.34 (0.23-0.44) | 0.45 (0.33-0.55) |
| AUROC | 0.84 (0.81-0.87) | 0.85 (0.81-0.89) | 0.78 (0.72-0.83) | 0.84 (0.77-0.89) |
| AUPRC | 0.29 (0.22-0.36) | 0.43 (0.33-0.54) | 0.27 (0.17-0.38) | 0.54 (0.41-0.67) |
| Calibration slope | 0.93 (0.81-1.06) | 1.10 (0.90-1.35) | 0.84 (0.64-1.07) | 0.99 (0.76-1.26) |
| Calibration intercept | -0.09 (-0.41-0.26) | 0.52 (0.03-1.06) | -0.26 (-0.83-0.34) | 0.54 (0.01-1.12) |
| Brier score | 0.05 (0.05-0.06) | 0.07 (0.06-0.09) | 0.06 (0.05-0.08) | 0.10 (0.08-0.12) |

##### **Supplementary Table 6.** Model performance by occurrence of out-of-hospital cardiac arrest (OHCA) and test cohort. Values are median (95% CI)

| **Metric\Subpopulation** | **OHCA Augsburg** | **OHCA Brandenburg** | **No OHCA Augsburg** | **No OHCA Brandenburg** |
| --- | --- | --- | --- | --- |
| **N samples** | **128** | **96** | **2614** | **1,205** |
| Sensitivity | 0.84 (0.72-0.94) | 0.85 (0.73-0.94) | 0.28 (0.19-0.36) | 0.32 (0.23-0.40) |
| Specificity | 0.50 (0.39-0.61) | 0.62 (0.48-0.76) | 0.96 (0.95-0.96) | 0.94 (0.93-0.95) |
| PPV | 0.47 (0.36-0.58) | 0.68 (0.55-0.79) | 0.23 (0.17-0.30) | 0.34 (0.25-0.43) |
| F1-score | 0.60 (0.50-0.70) | 0.75 (0.65-0.84) | 0.25 (0.18-0.32) | 0.33 (0.24-0.41) |
| AUROC | 0.68 (0.58-0.77) | 0.79 (0.68-0.88) | 0.80 (0.76-0.84) | 0.81 (0.77-0.85) |
| AUPRC | 0.47 (0.34-0.61) | 0.71 (0.56-0.86) | 0.19 (0.13-0.26) | 0.31 (0.23-0.40) |
| Calibration slope | 0.57 (0.29-0.89) | 0.89 (0.48-1.44) | 0.93 (0.78-1.08) | 0.98 (0.81-1.17) |
| Calibration intercept | -0.09 (-0.57-0.42) | 0.86 (0.23-1.64) | -0.30 (-0.72-0.12) | 0.25 (-0.22-0.72) |
| Brier score | 0.22 (0.18-0.26) | 0.22 (0.18-0.28) | 0.04 (0.03-0.05) | 0.07 (0.06-0.08) |

##### **Supplementary Table 7.** Model performance by treatment strategy and test cohort. Values are median (95% CI)

| **Metric\Subpopulation** | **Invasive Treatment Augsburg** | **Invasive Treatment Brandenburg** | **Conservative Treatment Augsburg** | **Conservative Treatment Brandenburg** |
| --- | --- | --- | --- | --- |
| **N samples** | **2399** | **1,152** | **546** | **111** |
| Sensitivity | 0.40 (0.31-0.49) | 0.45 (0.35-0.55) | 0.42 (0.32-0.52) | 0.44 (0.19-0.69) |
| Specificity | 0.95 (0.94-0.95) | 0.94 (0.92-0.95) | 0.89 (0.86-0.92) | 0.89 (0.81-0.95) |
| PPV | 0.26 (0.20-0.33) | 0.43 (0.34-0.53) | 0.45 (0.34-0.56) | 0.39 (0.17-0.62) |
| F1-score | 0.31 (0.24-0.39) | 0.44 (0.36-0.52) | 0.43 (0.34-0.52) | 0.41 (0.19-0.60) |
| AUROC | 0.83 (0.79-0.87) | 0.84 (0.79-0.87) | 0.76 (0.70-0.81) | 0.79 (0.65-0.89) |
| AUPRC | 0.22 (0.16-0.29) | 0.39 (0.30-0.49) | 0.40 (0.30-0.51) | 0.50 (0.25-0.70) |
| Calibration slope | 0.92 (0.78-1.06) | 1.01 (0.84-1.21) | 0.75 (0.56-0.95) | 0.84 (0.39-1.32) |
| Calibration intercept | -0.45 (-0.84--0.07) | 0.31 (-0.11-0.79) | 0.21 (-0.27-0.72) | 0.18 (-0.96-1.12) |
| Brier score | 0.04 (0.03-0.05) | 0.07 (0.06-0.08) | 0.13 (0.11-0.15) | 0.11 (0.06-0.15) |

## Missing data and sensitivity analyses

##### **Supplementary Table 8.** Comparison of patients with documented and unknown 28-day mortality status on Brandenburg registry. Quantitative values are presented as median (95%-CI; total entries) and categorical variables as count/total entries (percentage).

|  | **Brandenburg**  **Documented 28-day mortality status** | **Brandenburg**  **Undocumented 28-day mortality status** | **p-value <0.05** |
| --- | --- | --- | --- |
| Patients | 1,328 | 304 | - |
| Males | *921/1,325 (69%)* | 207/303 (68%) | 0.1 |
| Age | *70 (45-91; 1,325)* | 65 (42-91; 300) | 0.06 |
| Hypertension | 991/1,304 (75%) | 204/303 (68%) | 0.001 |
| Hyperlipidaemia | 572/1,228 (46%) | 103/261 (39%) | <0.0001 |
| Active smoker | 560/906 (61%) | 119/165 (72%) | <0.0001 |
| Previous stroke | 88/566 (15%) | 13/100 (13%) | 0.2 |
| Diabetes | 440/1,306 (33%) | 78/297 (26%) | 0.02 |
| Out-of-hospital cardiac arrest | *96/1,301 (7%)* | 16/293 (5%) | <0.0001 |
| Angina Pectoris | 1,130/1,306 (86%) | 249/294 (84%) | <0.0001 |
| Dyspnea | 695/1,301 (53%) | 167/292 (57%) | 0.001 |
| Sweating | 268/1,328 (20%) | 75/304 (24%) | <0.0001 |
| Syncope | 103/1,307 (7%) | 18/292 (6%) | 0.9 |
| Heart rate at admission (bpm) | *84(50-140; 1,268)* | **84 (50-130; 281)** | <0.0001 |
| Systolic blood pressure at admission (mmHg) | *146 (85-199; 1,252)* | 144 (90-199; 282) | 0.3 |
| Diastolic blood pressure at admission (mmHg) | *84 (50-122; 1,252)* | 84 (53-118; 281) | 0.0003 |
| Positive Troponin | *1,256/1,326 (94%)* | 285/301 (94%) | 0.0002 |
| C-Reactive Protein (mg/dL) | *0.46 (0.06-16.79; 1,312)* | 0.47 (0.06-15.06; 302) | 0.8 |
| Glucose (mmol/L) | *7.9 (4.9-20.4; 1,282)* | 7.8 (5.2-21.8; 298) | 0.001 |
| Creatinine (mg/dL) | *1.0 (0.6-2.8; 1,319)* | 0.97 (0.6-2.1; 301) | 0.008 |
| Total Cholesterol (mg/dL) | *180 (93-296; 944)* | 191 (100-289; 220) | 0.1 |
| Leucocytes (/nL) | *9.9 (5.3-21.2, 1,324)* | 10.7 (4.9-21.9; 303) | 0.03 |
| STEMI | 451/1,323 (34%) | 112/302 (37%) | 0.9 |
| Anterior wall MI | 583/1,223 (47%) | 142/267 (53%) | 0.5 |
| Conservative Treatment | 111/1,263 (8%) | 23/281 (8%) | <0.0001 |
| PCI | 1,074/1,262 (85%) | 234/281 (83%) | <0.0001 |
| Aort-coronary Bypass | 86/1,253 (6%) | 32/279 (11%) | 0.2 |
| Left Ventricular Ejection Fraction (LVEF)<30% | 129/1,138 (11%) | 28/261 (10%) | <0.0001 |
| Cardiogenic shock | 82/211(38%) | 9/20 (45%) | <0.0001 |
| In hospital Stroke | 5/205(2%) | 0/20 (0%) | 1 |
| In hospital Bleeding | 26/209 (12%) | 8/20 (40%) | <0.0001 |
| 28-day mortality | 153/1,328 (11%) | - | - |

##### **Supplementary Table 9.** Sensitivity analysis if all Brandenburg patients with unknown 28-day mortality either all survived or died.

| **Metric** | **All patients without follow-up survive** | **All patients without follow-up die** |
| --- | --- | --- |
| Sensitivity | 0.49 (0.41-0.57) | 0.21 (0.18-0.25) |
| Specificity | 0.93 (0.91-0.94) | 0.93 (0.91-0.94) |
| PPV | 0.41 (0.34-0.48) | 0.54 (0.47-0.61) |
| F1-score | 0.45 (0.38-0.51) | 0.31 (0.26-0.35) |
| AUROC | 0.85 (0.81-0.88) | 0.61 (0.57-0.64) |
| AUPRC | 0.41 (0.33-0.49) | 0.43 (0.38-0.48) |
| Slope | 1.04 (0.90-1.21) | 0.33 (0.24-0.42) |
| Intercept | 0.25 (-0.10-0.62) | 0.05 (-0.22-0.31) |
| Brier score | 0.07 (0.06-0.08) | 0.23 (0.21-0.25) |

## Sensitivity to imputation

##### **Supplementary Table 10.** Proportion of missing values for candidate predictors in the Augsburg and Brandenburg cohorts.

|  | **Augsburg** | **Brandenburg** |
| --- | --- | --- |
| **Male sex** | 0 | 0.23% |
| **Age** | 0 | 0.22% |
| **Out-of-hospital cardiac arrest** | 6.76% | 2.03% |
| **Heart rate at admission (bpm)** | 2.69% | 4.51% |
| **Systolic blood pressure at admission (mmHg)** | 3.24% | 5.72% |
| **Diastolic blood pressure at admission (mmHg)** | 5.86% | 5.72% |
| **Positive Troponin** | 38.64% | 0.15% |
| **C-Reactive Protein (mg/dL)** | 7.13% | 1.20% |
| **Glucose (mmol/L)** | 4.54% | 3.46% |
| **Creatinine (mg/dL)** | 22.83% | 0.67% |
| **Total Cholesterol (mg/dL)** | 63.18% | 28.91% |
| **Leucocytes (/nL)** | 22.64% | 0.30% |
| **STEMI** | 4.88% | 0.38% |
| **Anterior wall MI** | 32.01% | 7.91% |

##### **Supplementary Table 11.** Discrimination and calibration metrics for the gradient boosting machine model using different imputation strategies for continuous variables (mode, median, mean). Values are reported as median (95% CI).

|  | **Augsburg Validation** | | | **Augsburg Test** | | | **Brandenburg** | | |
| --- | --- | --- | --- | --- | --- | --- | --- | --- | --- |
| **Discrimination** | GBM+mode | GBM+median | GBM+mean | GBM+mode | GBM+median | GBM+mean | GBM+mode | GBM+median | GBM+mean |
| Sensitivity | 0.48 (0.36-0.58) | 0.41 (0.34-0.47) | 0.38 (0.32-0.45) | 0.48 (0.42-0.55) | 0.49 (0.41-0.57) | 0.48 (0.40-0.56) | 0.55 (0.47-0.63) | 0.48 (0.36-0.58) | 0.41 (0.34-0.47) |
| Specificity | 0.93 (0.92-0.95) | 0.94 (0.93-0.95) | 0.95 (0.94-0.96) | 0.92 (0.91-0.93) | 0.93 (0.91-0.94) | 0.93 (0.92-0.95) | 0.91 (0.90-0.93) | 0.93 (0.92-0.95) | 0.94 (0.93-0.95) |
| PPV | 0.34 (0.25-0.42) | 0.32 (0.27-0.38) | 0.35 (0.29-0.42) | 0.31 (0.27-0.37) | 0.47 (0.39-0.54) | 0.48 (0.41-0.56) | 0.45 (0.38-0.52) | 0.34 (0.25-0.42) | 0.32 (0.27-0.38) |
| F1-score | 0.39 (0.31-0.48) | 0.36 (0.31-0.42) | 0.37 (0.31-0.43) | 0.38 (0.33-0.43) | 0.48 (0.41-0.55) | 0.48 (0.41-0.55) | 0.50 (0.43-0.56) | 0.39 (0.31-0.48) | 0.36 (0.31-0.42) |
| AUROC | 0.87 (0.83-0.91) | 0.82 (0.79-0.85) | 0.84 (0.82-0.87) | 0.85 (0.82-0.87) | 0.85 (0.81-0.88) | 0.83 (0.79-0.86) | 0.83 (0.80-0.87) | 0.87 (0.83-0.91) | 0.82 (0.79-0.85) |
| AUPRC | 0.33 (0.24-0.44) | 0.28 (0.22-0.34) | 0.29 (0.23-0.35) | 0.31 (0.25-0.38) | 0.47 (0.39-0.55) | 0.45 (0.37-0.54) | 0.46 (0.38-0.54) | 0.33 (0.24-0.44) | 0.28 (0.22-0.34) |
| **Calibration** |  |  |  |  |  |  |  |  |  |
| Slope | 0.95 (0.79-1.13) | 0.91 (0.80-1.02) | 0.87 (0.77-0.99) | 0.88 (0.78-0.99) | 1.07 (0.92-1.24) | 0.89 (0.75-1.04) | 0.92 (0.79-1.07) | 0.95 (0.79-1.13) | 0.91 (0.80-1.02) |
| Intercept | -0.01 (-0.45-0.46) | -0.13 (-0.41-0.17) | -0.19 (-0.47-0.12) | -0.19 (-0.46-0.09) | 0.55 (0.19-0.93) | 0.23 (-0.10-0.56) | 0.29 (-0.04-0.63) | -0.01 (-0.45-0.46) | -0.13 (-0.41-0.17) |
| Brier score | 0.05 (0.04-0.06) | 0.06 (0.05-0.06) | 0.06 (0.05-0.06) | 0.05 (0.05-0.06) | 0.08 (0.07-0.09) | 0.08 (0.07-0.09) | 0.08 (0.07-0.09) | 0.05 (0.04-0.06) | 0.06 (0.05-0.06) |

# References

1. Killip, T. & Kimball, J. T. Treatment of myocardial infarction in a coronary care unit: A Two year experience with 250 patients. *Symp. Coron. Care Units* **20**, 457–464 (1967).
